# Unveiling the etiology of peritonsillar abscess using next generation sequencing

**DOI:** 10.1186/s12941-023-00649-0

**Published:** 2023-11-08

**Authors:** Merili Saar, Risto Vaikjärv, Ülle Parm, Priit Kasenõmm, Siiri Kõljalg, Epp Sepp, Madis Jaagura, Andres Salumets, Jelena Štšepetova, Reet Mändar

**Affiliations:** 1 Department of Microbiology, Institute of Biomedicine and Translational Medicine, Faculty of Medicine, University of Tartu, Ravila 19, Tartu, 50411 Estonia; 2 Competence Center on Health Technologies, Tartu, Estonia; 3 Ear-Nose-Throat Clinic, Tallinn, Estonia; 4 Tartu Health Care Colleges, Tartu, Estonia; 5 Ear Clinic, Tartu University Hospital, Tartu, Estonia; 6 Department of Oto-Rhino-Laryngology, Institute of Clinical Medicine, University of Tartu, Tartu, Estonia; 7 Laboratory of Clinical Microbiology, United Laboratories, Tartu University Hospital, Tartu, Estonia; 8 Institute of Genomics, University of Tartu, Tartu, Estonia; 9 Division of Obstetrics and Gynecology, Department of Clinical Science, Intervention and Technology, Karolinska Institutet and Karolinska University Hospital, Stockholm, Sweden; 10 Department of Obstetrics and Gynaecology, Institute of Clinical Medicine, University of Tartu, Tartu, Estonia

**Keywords:** Peritonsillar abscess (PTA), Microbiota, *Streptococcus pyogenes*, *Fusobacterium necrophorum*, *Fusobacterium nucleatum*, Next generation sequencing (NGS)

## Abstract

**Background:**

Peritonsillar abscess (PTA) is a severe deep neck space infection with an insufficiently characterized bacterial etiology. We aimed to reveal the bacteria associated with PTA applying next generation sequencing (NGS). Tonsil biopsies and pus samples of 91 PTA patients were analysed applying NGS method.

**Results:**

Over 400 genera and 800 species belonging to 34 phyla were revealed. The most abundant species in both sample types were *Streptococcus pyogenes, Fusobacterium necrophorum* and *Fusobacterium nucleatum*. When present, *S. pyogenes* was normally a predominant species, although it was recovered as a minor population in some samples dominated by *F. nucleatum* and occasionally *F. necrophorum*. *S. pyogenes* and *F. necrophorum* were the predominant species (> 10% in a community) in 28 (31%) pus samples, while *F. nucleatum* in 21 (23%) and *S. anginosus* in 8 (9%) pus samples. We observed no substantial differences between the microbial findings in pus and tonsil biopsies.

**Conclusions:**

The most probable causative agents of PTA according to our NGS-study include *Streptococcus pyogenes, Fusobacterium necrophorum* and *Fusobacterium nucleatum.* Some other streptococci (*S. anginosus*) and anaerobes (*Prevotella*, *Porphyromonas*) may contribute to the infection as well. Pus of the peritonsillar abscess is more representative specimen for microbiological examination than the tonsillar tissue. Our results are important in the context of optimizing the handling of the PTA patients.

**Supplementary Information:**

The online version contains supplementary material available at 10.1186/s12941-023-00649-0.

## Background

Peritonsillar abscess (PTA) is a collection of pus between the tonsil fibrous capsule and superior pharyngeal constrictor muscle. It is the most frequent cause of acute admissions in otorhinolaryngology [1–4]. Although it can occur at any age, the highest incidence has been shown between years 15 to 40 [2, 3]. PTA is considered a suppurative complication of acute tonsillitis, where bacteria from the tonsillar mucosa invade the surrounding tissue [1, 2]. It has been proposed that in some cases, the abscess may originate from Weber’s salivary glands [5] or that minor salivary glands are sometimes involved in the development of PTA [6]. Rising amounts of evidence associate PTA with periodontal disease and smoking [7]. However, the true pathophysiology of the disease is still widely unclear. When untreated, severe complications may occur, such as descending mediastinitis, para- and retropharyngeal abscess, and necrotizing fasciitis [3]. Thus, rapid diagnosis, usually based on clinical and physical examination, and fast-acting treatment is required [2]. The latter involves mostly a surgical intervention, such as needle aspiration, incision and drainage or acute tonsillectomy, with the addition of antimicrobial therapy and acute pain management [1–3].

The spectrum of microbes involved in the infection remains poorly characterized. Previous findings are greatly varying, and cultures obtained from PTA materials are frequently polymicrobial [3, 8]. Although *Streptococcus pyogenes* is currently considered to be the main causative agent of PTA, it is only recovered from approximately one third of PTA specimens [8–11]. Recent studies have also investigated the role of *Fusobacterium necrophorum* (FN) in PTA, which is found in about a quarter of PTA materials using DNA-based identification [8]. A significant increase in anti-FN antibody levels was observed in PTA patients with FN-positive pus aspirate cultures [9], providing a strong basis to include FN in the list of microbes responsible for the development of PTA. Other microbes, such as viridans-streptococci, *Streptococcus anginosus* group (SAG), *Staphylococcus aureus*, *Bacteroides* spp., *Prevotella* spp., *Neisseria* spp., and *Klebsiella pneumoniae* might also occasionally play a role in the development of PTA [3, 10, 12]. However, no previous studies have applied next generations sequencing (NGS) to obtain a complete and unbiased description of bacteria in PTA-samples.

The optimal specimen type for microbial diagnostics of PTA remains undetermined. Both healthy and diseased tonsils are heavily colonized with bacteria, complicating routine diagnostics as well as scientific investigations. In a previous study we compared culture of pus aspired from the abscess to culture of tissue biopsies from the tonsillar fossa, showing that the latter revealed more bacterial species per sample [13]. As the main function of tonsils is to prevent infections with activated B and T lymphocytes after contact with harmful bacteria [14], we assumed that it might be the place to search for the microbes causing the disease.

As there are no commonly accepted guidelines for the treatment of PTA, handling of the patients varies greatly between countries, hospitals and physicians and depend a lot on the physician’s experience and intuition. The initial antibiotic therapy usually includes broad-spectrum antimicrobial therapy targeted against streptococci and oral anaerobes [2, 3, 8, 15]. Reliable knowledge about the most probable pathogens and about the most optimal type of specimen would improve the handling of PTA patients significantly.

We aimed to detect the microbes present in both tonsils and peritonsillar pus of PTA patients applying NGS in order to reveal the most probable causative agents of PTA. We also aimed to define the best specimen type for their detection.

## Materials and methods

### Patient cohort

A prospective study was performed between February 2016 and January 2019 at the Ear Clinic, Tartu University Hospital, Estonia. The patient cohort included 91 PTA patients (53 male and 38 female patients, with median age of 31.5, range 13−67 years) undergoing bilateral tonsillectomy due to PTA. Exclusion criteria were age younger than 12 years, diabetes type I and II, current administration of immunosuppressants, immunosuppressive disease, and current chemo- or radiation therapy. The patient cohort was described more thoroughly elsewhere [16].

The study was approved by the Ethics Review Committee on Human Research of the University of Tartu (Permission No. 255/T-1). Participation in the study was voluntary. All patients were included after their written informed consent was obtained.

### Clinical handling of the patients and specimen collection

The diagnosis of PTA was based on clinical evaluation prior to surgery and the presence of pus in the peritonsillar space. Basic clinical data were recorded (Table 1). Blood indices were examined in the United Laboratory of Tartu University Hospital by using standard methods.

**Table 1:** Clinical and blood variables in study subjects

Clinical data	Mean ± SD	Median (range)
Age (years)	33.5 ± 13.8	31.5 (13–67)
Antibiotic treatment before hospitalization	n = 45 (49.5%) *	
Body temperature (°C)	37.6 ± 0.6	37.5 (36.0–39.4)
Duration of symptoms before hospitalization (days)	5.5 ± 4.1	5 [1–30)
C-reactive protein (mg/L)	100.1 ± 67.4	86.9 (6.9–334.4)
WBC (x 10^9^/L)	13.6 ± 3.7	13.8 (6.8–22.7)
Anti-streptolysin O (IU/mL)	259.9 ± 405.3	147.0 (20.0–3079.0)

* data is given as number (percentage) of patients

All surgical procedures were performed within 24 h of admission under general anaesthesia. The tonsils were removed by blunt dissection, followed by collection of pus by swabbing the abscess cavity. Both the pus sample and the removed tonsil were stored in sterile tubes at -80 ^o^C until DNA extraction.

### Molecular analyses

Bacterial DNA from washouts of the swabs was extracted with the PureLinkTM Microbiome DNA Purification Kit (Invitrogen, USA), using the ELMI Sky Line instrument (ELMI Ltd, Riga, Latvia) according to manufacturer’s instructions. The frozen tonsil biopsy specimens (~ 25 mg) were suspended in 500 µL of lysis buffer (200 mM Tris-HCl (pH 8.0), 25 mM ethylenediaminetetraacetic acid (EDTA), 300 mM NaCl, 1.2% sodium dodecyl sulfate) and 20 µL of proteinase K (400 µg/mL) for DNA extraction. The mixture was incubated at 37 ◦C for 24 h. The procedure of DNA extraction was continued according to the tissue protocol of QIAamp DNA Mini Kit (Qiagen, Hilden, Germany). DNA was amplified within the hypervariable (V3-V4) region of prokaryotic 16 S ribosomal RNA gene. Sequencing was carried out on an Illumina MiSeq System using MiSeq Reagent Kit v3 in paired end 2 × 300 bp mode. Details of molecular methods are presented in Table S1.

### Statistical analysis

The statistical analysis was performed using SigmaPlot 14.5 (Systat Software, Inc., USA). Statistical significance was determined using descriptive statistics, Mann-Whitney, chi-square, and Spearman correlation tests. The level of statistical significance was considered to be p < 0.05. R software (R version 4.1.1, R, Vienna, Austria) was used for generation of figures.

## Results

### Clinical characteristics of the patient cohort

Specimens from 91 PTA patients were studied (Table 1). There was a significant male patient predominance (53 out of 91; p = 0.038), and nearly half of the patients (49.5%) had used antibiotics prior to hospitalization, including amoxicillin with clavulanic acid (21%), penicillin V (19%), amoxicillin (19%), clarithromycin (15%), cefadroxil (6%), cefuroxime (6%), clindamycin (4%), cefprozil (4%), azithromycin (2%) and ciprofloxacin (2%). Blood analyses revealed increased levels of WBC, C-reactive protein, and anti-streptolysin O. One fifth of the patients were regular smokers. Age, smoking and antibiotic use did not have impact on microbial communities (Figure S1). Additional information about the cohort has been published previously [16].

### Overall sequence statistics and diversity analysis

**Fig. 1:**
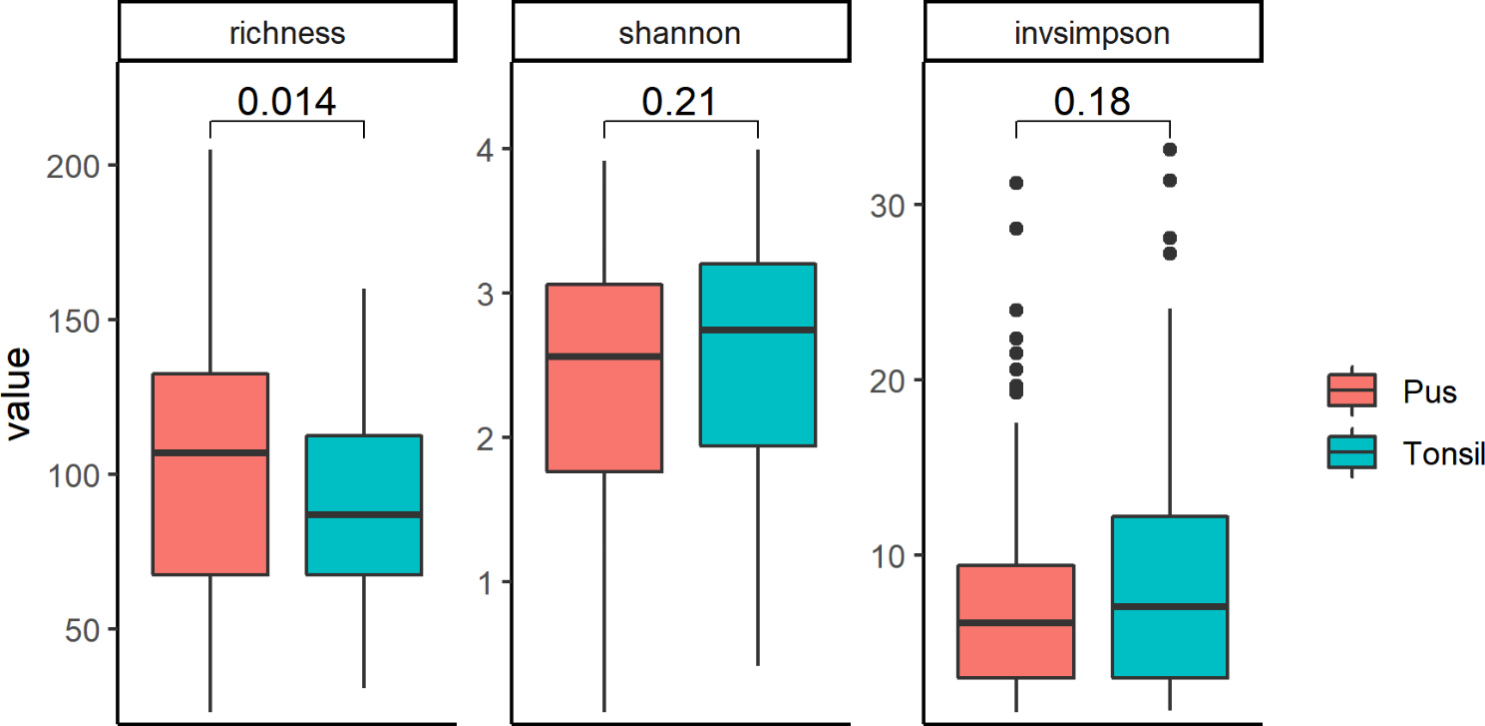
Species richness, Shannon’H’ diversity and simpson indeces in the tonsillar biopsy (blue) and pus (red) specimens

**Fig. 2:**
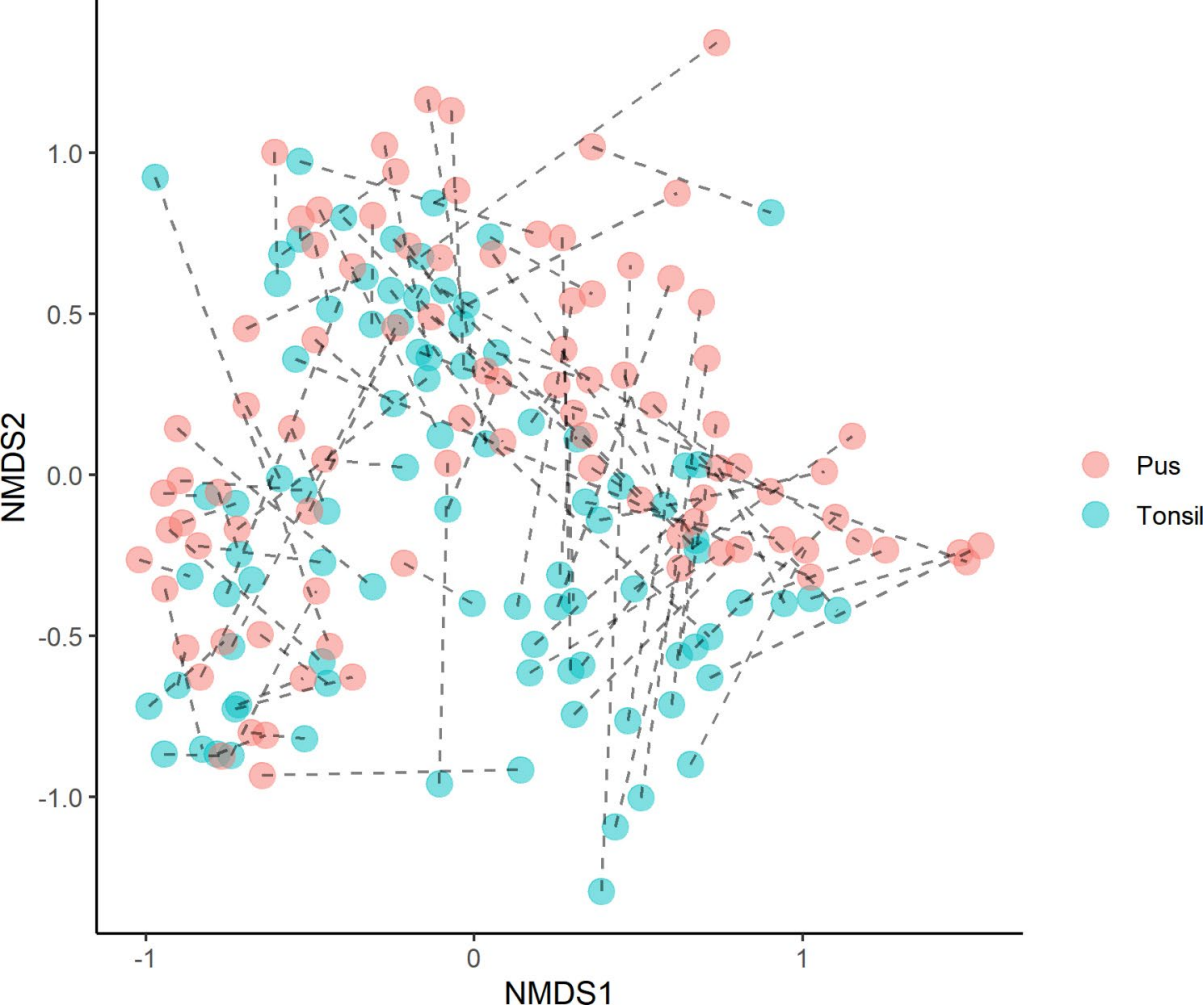
Non-metric multidimensional scaling (NMDS) plots showing the difference of pus (red) and tonsillar biopsy (blue) microbiota across elevations at species level based on beta-diversity index (Bray-Curtis similarity index). Samples of one patient are connected by a dotted line

A total of 5,058,572 high quality reads were obtained, with a mean of 19 475 reads per tonsil and 36 114 per pus sample. The OTUs were classified into 34 phyla, 476 genera, 880 species and 331 unclassified groups. Shannon ’H’ diversity (proportion of the species) and Simpson’s indices (number of the species/relative abundance of the species) showed no significant difference between the sample types, while species richness (number of species in a community) was significantly higher in pus than in tonsils (mean value of 104±43 and 86±28, respectively) (Fig. 1). The beta-diversity analysis revealed a clear separation between pus samples and tonsil biopsies. Additionally, clustering between pus and tonsil biopsy of the same patient was demonstrated (Fig. 2).

### Microbial communities in tonsils

The incidences for the most predominant bacteria in tonsil biopsies is provided in Table 2, and their main relative abundances are shown in Fig. 3. The incidence and relative abundance for all detected bacteria are listed in **Tables S2-S5**. Nearly one third of an average tonsil microbiota was composed of the Gram-negative anaerobic **genera***Fusobacterium* and *Prevotella* (mean relative abundances 22.2 and 13.4%, respectively) while the genus *Streptococcus* had a mean relative abundance of 14.3%. At the **species** level, *Fusobacterium necrophorum* displayed the highest mean relative abundance (14.6%), followed by *Streptococcus pyogenes* (10%) and *Fusobacterium nucleatum* (6.8%). Other species displaying a high mean relative abundance in tonsils were *Prevotella oris* (4%) and *Porphyromonas endodontalis* (3.1%).

**Table 2:** Incidence of the predominant bacteria* in pus and tonsil tissue of PTA patients

Phylum	Incidence (%) in		Genus	Incidence (%) in		Species	Incidence (%) in	
	Pus	Tonsil		Pus	Tonsil		Pus	Tonsil
*Actinobacteriota*	99	100						
*Bacteroidota*	100	100	*Prevotella*	98	100	*Prevotella oris*	91	87
			*Porphyromonas*	95	90	*Porphyromonas endodontalis*	76	70
			*Alloprevotella*	95	92			
			*Chryseobacterium*	38	97	*Chryseobacterium hominis*	16	97
*Cyanobacteria*	47	59						
*Firmicutes*	100	100	*Streptococcus*	100	99	*Streptococcus pyogenes*	70	66
						*Streptococcus mitis*	74	82
						*Streptococcus anginosus*	89	72
			*Veillonella*	91	95			
			*Parvimonas*	90	86	*Parvimonas micra*	90	84
			*Peptostreptococcus*	77	73	*Peptostreptococcus stomatis*	77	72
*Fusobacteriota*	99	100	*Fusobacterium*	98	100	*Fusobacterium necrophorum*	75	82
						*Fusobacterium nucleatum*	86	89
*Proteobacteria*	100	100	*Haemophilus*	95	88	*Haemophilus parainfluenzae*	89	90
			*Sphingomonas*	32	97	*Sphingomonas faeni*	5	97
			*Allorhizobium* #	36	99			
*Patescibacteria*	78	67						
*Spirochaedota*	78	77	*Treponema*	78	77			
*Synergistota*	48	51						

* Table displays the incidence of the bacteria whose mean relative abundance is given on Fig. 3

# *Allorhizobium-Neorhizobium-Pararhizobium-Rhizobium* group

**Fig. 3:**
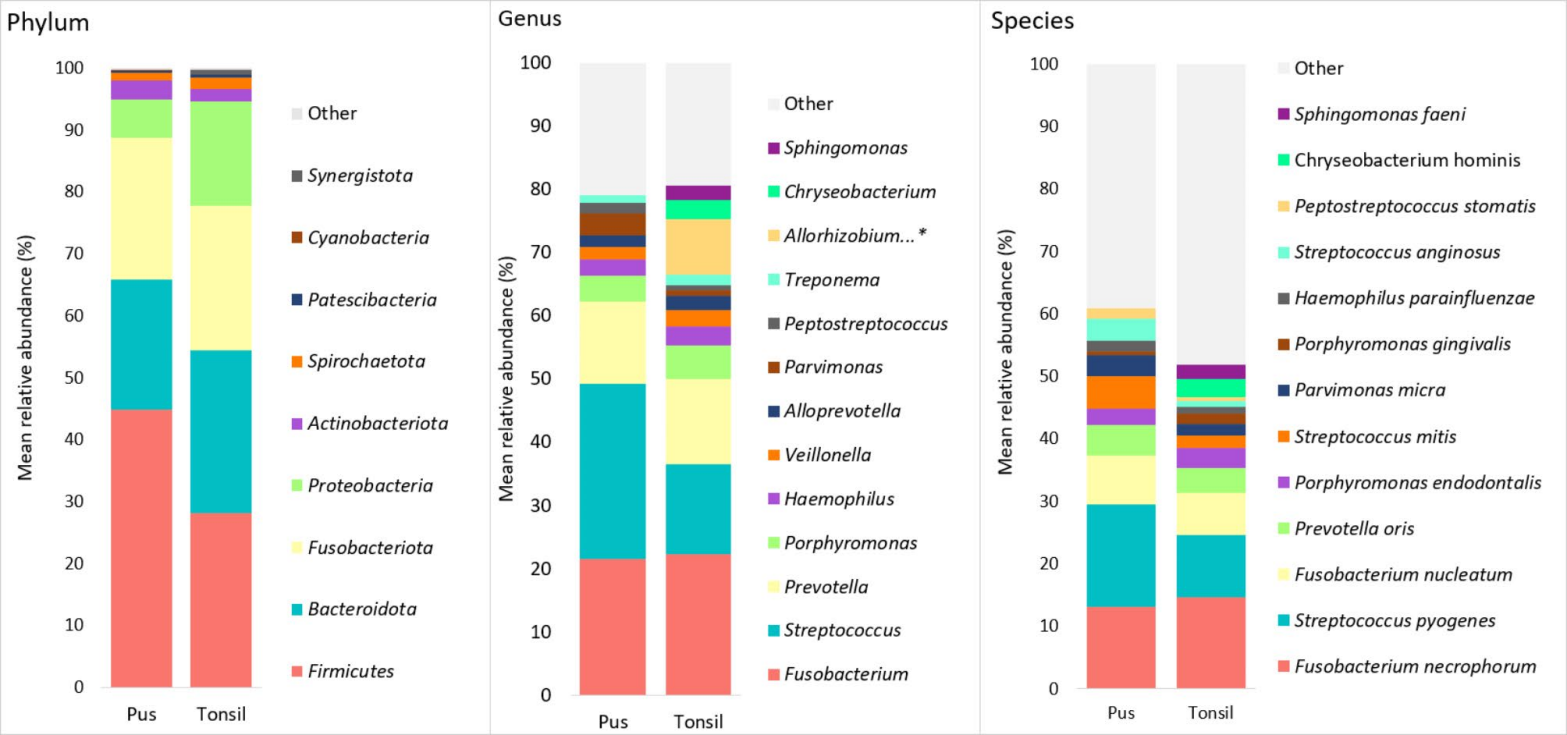
Microbial composition of tonsils and pus on phylum, genus and species level according to mean relative abundance. On genus and species level, top 10 bacterial taxa from both sample types are shown. Compared to pus, *Bacteroidota* and *Proteobacteria* relative abundances were significantly higher in tonsils (p = 0.038 and < 0.001, respectively), while pus samples contained more *Firmicutes* and *Actinobacteria* (p < 0.001 and 0.017, respectively). * - *Allorhizobium-Neorhizobium-Pararhizobium-Rhizobium* group

### Microbial communities in pus

The incidences for the most predominant bacteria in pus samples is provided in Table 2, and their main relative abundances are shown in Fig. 3. On the **genus** level, streptococci were present in all pus samples and had the highest mean relative abundance (27.6%). Fusobacteria represented the second most dominant genus with a mean relative abundance of 21.6%. In contrast to the tonsil samples, the highest mean relative abundancies at the **species** level was found for *S. pyogenes* (16.7%), followed by *F. necrophorum* (13.3%) and *F. nucleatum* (7.8%). *Streptococcus mitis* and *P. oris* were also present in slighlty higher quantities than in tonsils (5.2 and 5.1%, respectively).

It is notable that anaerobic bacteria were highly abundant in the majority of pus samples, constituting more than 10% in 59 (15 with and 44 without *S. pyogenes*) out of 91 samples and more than 50% in 25 samples (**Figure S2**).

### Microbial comparison of pus and tonsil samples

At the **genus** level, the overall bacterial composition of the two sample types were similar. Both pus and tonsillar biopsies were dominated by streptococci or fusobacteria, and with smaller quantities of *Prevotella*. Concerning environmental and/or non-pathogenic bacteria, tonsils displayed significantly higher levels of the genera *Sphingomonas*, *Chryseobacterium*, and bacteria from the *Allorhizobium-Neorhizobium-Pararhizobium-Rhizobium* group (all p < 0.001). Pus samples displayed significantly more streptococci (p < 0.001), *Gemella* (p = 0.02) and *Actinomyces* (p = 0.007).

**Species**-level analysis revealed a very diversely colonised environment in both tonsils and pus, and there were no species that would be present in all studied samples. Predominant species in both specimen types were *S. pyogenes, F. necrophorum* and *F. nucleatum*. In pus samples, *Streptococcus anginosus* (p = 0.012), *Granulicatella adiacens* (p = 0.007), and *Eubacterium brachy* (p = 0.012) were found to present significantly higher incidence and abundance than in tonsils. Tonsils displayed a significantly higher level of non-pathogenic bacteria in terms of both incidence and relative abundances, such as *C. hominis* and *S. faeni* (both p < 0.001) than pus.

### Evaluation of predominant bacteria in individual samples

**Fig. 4:**
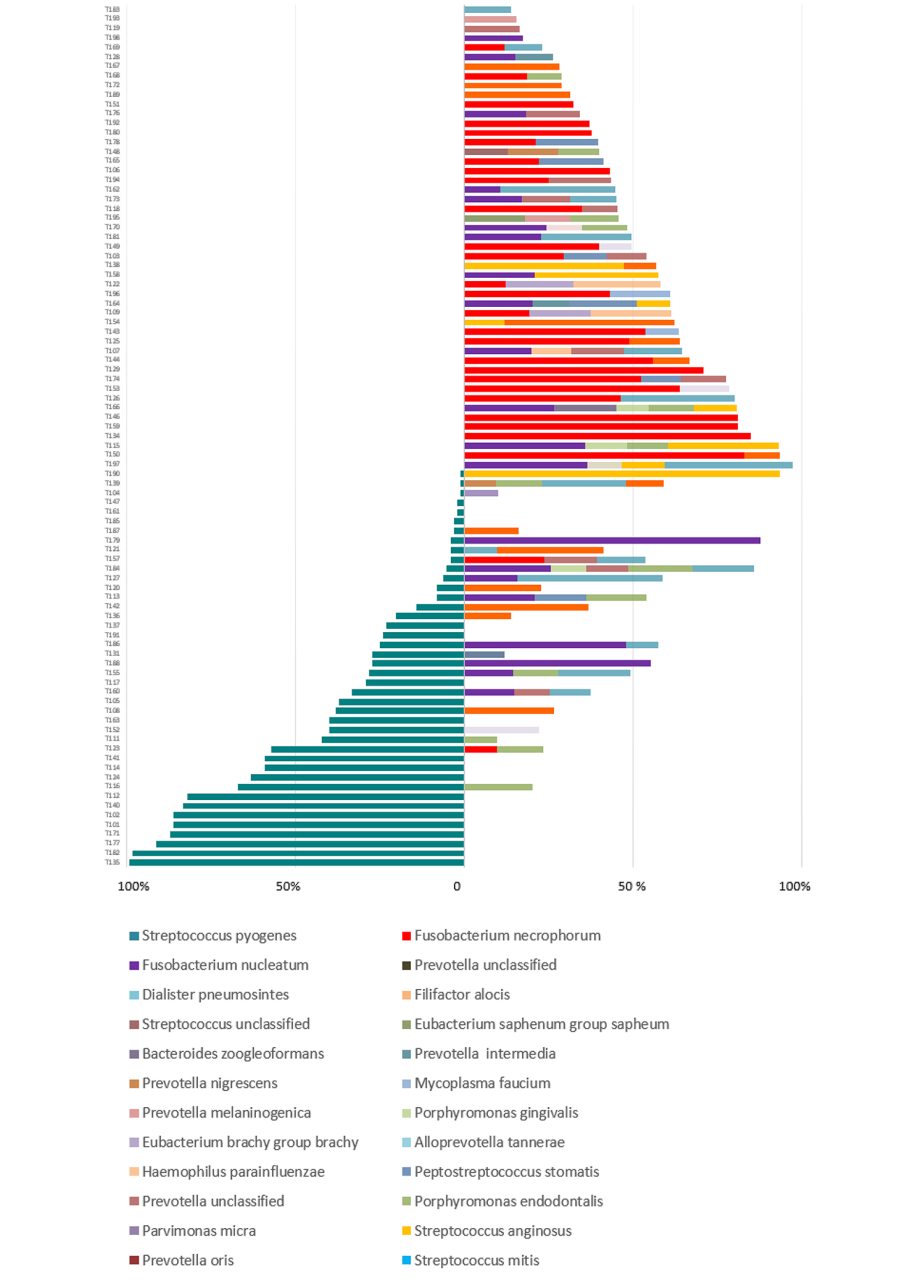
Relative distribution of the bacteria in the pus of peritonsillar abscess. Left side indicates only *S. pyogenes*, irrespective of the bacterial count, right side indicates the other bacteria having proportion over 10%

Figure 4 illustrates the relative distributions of *S. pyogenes*, fusobacteria and other predominant bacteria (having proportion over 10%) in pus samples of each particular patient. *S. pyogenes*, when present, was mostly as the predominant species. In cases of lower proportion (under 40%), *S. pyogenes* was often accompanied by high amounts of *F. nucleatum* (8 cases), but seldom with *F. necroforum*. The latter was found as the predominant species in half of non-*S. pyogenes* cases while *F. nucleatum* predominated in 13 and *Streptococcus anginosus* in 8 non-*S. pyogenes* cases. Other streptococci (*S. mitis* and unclassified streptococci) belonged among the predominant bacteria in a portion of pus samples both in the presence and absence of *S. pyogenes*. In addition, the anaerobic Gram negative rods *Porphyromonas* and *Prevotella* belonged among the most abundant bacteria in some pus samples. In 25 pus samples, a predominant species exceeded 50% of the total bacterial community, including 13 cases of *S. pyogenes*, 9 cases of *F. necrophorum*, 2 cases of *F. nucleatum*, and 1 case of *S. anginosus*.

It is notable that in three pus samples containing very small amounts of *S. pyogenes*, no microbe had a proportion above 10%. In these outlier samples with such large bacterial diversities, saliva contamination of the samples cannot be ruled out.

### Associations between the most predominant bacterial species

**Fig. 5:**
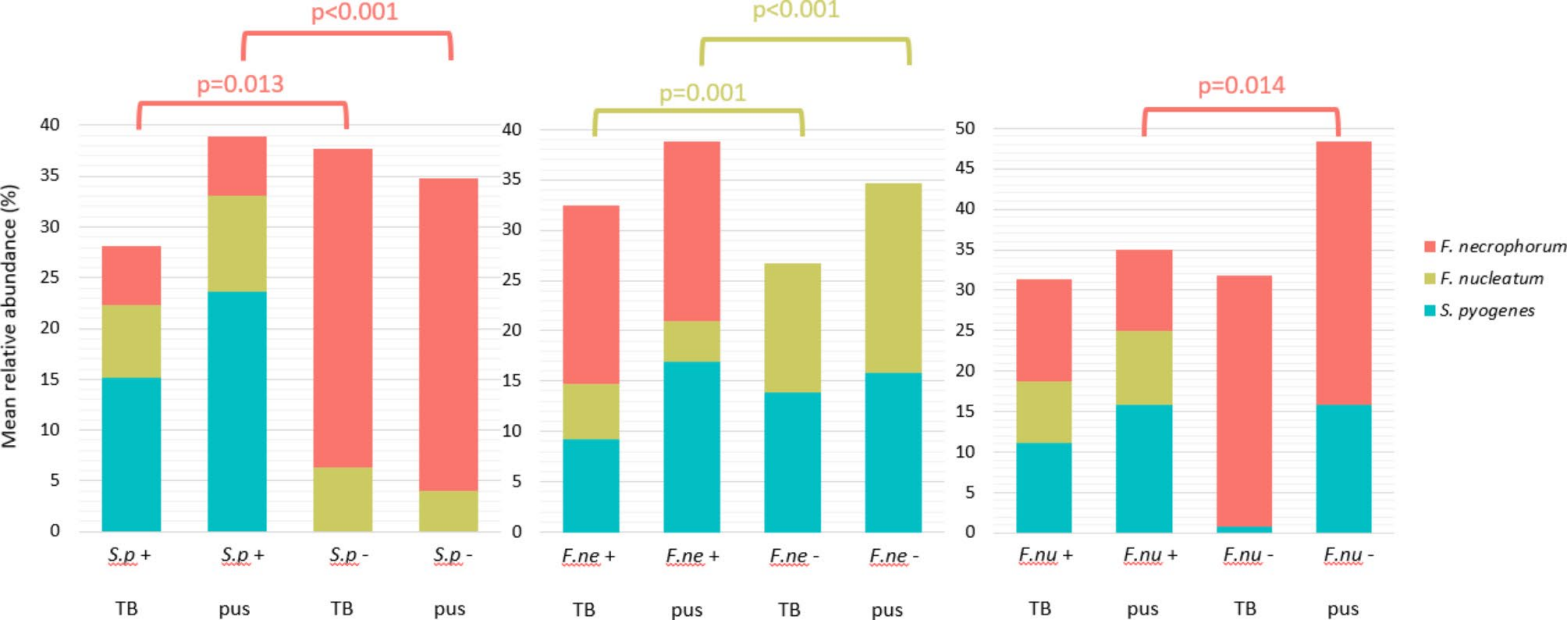
Mean relative abundance of the three suggested pathogens of peritonsillar abscess on the occasion where one or another suggested microbe is absent from the sample. **A** - Mean relative abundances of *F. necrophorum* and *F. nucleatum* in cases where *S. pyogenes* is present and where it is absent from the samples. **B** - Mean relative abundances of *S. pyogenes* and *F. nucleatum* in cases where *F. necrophorum* is present and where it is absent from the samples. **C -** Mean relative abundances of *S. pyogenes* and *F. necrophorum* in cases where *F. nucleatum* is present and where it is absent from the samples. Only the statistically significant *p* values are displayed on the figure. S.p, *Streptococcus pyogenes*; F.ne, *Fusobacterium necrophorum*; F.nu, *Fusobacterium nucleatum*; TB, tonsillar biopsy

We investigated the relationships between the three most predominant species in both specimen types by comparing their mean relative abundancies in the presence and absence of each of the two other species (Fig. 5). Samples without *S. pyogenes* contained high relative abundancies of *F. necrophorum*, while the samples with *S. pyogenes* displayed lower levels (less than 6%) of *F. necrophorum*, in both tonsils and pus. The presence of *F. necrophorum* was associated with lower relative abundancies of *F. nucleatum* in both sample types.

### Correlations between bacteria and clinical markers

Correlations between bacteria and clinical indices were seeked in case of both specimens. The proportion of *F. nucleatum* in the tonsils increased by age (R = 0.331, p = 0.002) while that of *F. necrophorum* decreased by age (R=-0.232, p = 0.029). Duration of symptoms before hospitalization was in positive association with *S. pyogenes*, both in tonsils (R = 0.279, p = 0.008) and pus (R = 0.296, p = 0.005). As expected, the level of anti-streptolysin O was in positive association with *S. pyogenes* (R = 0.275, p = 0.009) in tonsils.

## Discussion

Our results suggest that the most probable PTA-causing pathogens are *Streptococcus pyogenes* and two species of fusobacteria, *Fusobacterium nucleatum* and *Fusobacterium necrophorum*. Some other streptococci (including *S. anginosus*) and some other Gram negative anaerobic rods (*Prevotella, Porphyromonas*) may contribute to the infection as well. *S. pyogenes* and *F. necrophorum* tend to cause the infection rather individually than in combination while in case of other bacteria, polymicrobial infection is more likely. It is important to point that these bacteria are members of the normal nasopharyngeal microbiota [9]. Therefore, in a large number of cases, they may remain undetected in routine diagnostics or it may not be clear which of the isolated microbes is the cause of the infection, thus, it is important to address the methodological challenges [17] as well as pathogenic potential of the suspected bacteria. Our analysis was based on the results of NGS, the new golden standard of microbiome studies, and our results may be important in the context of optimizing the handling of the PTA patients.

***Streptococcus pyogenes*** is an acknowledged causative agent of tonsillitis and also PTA. In previous studies, *S. pyogenes* has been isolated in about 20–40% of PTA patients [3, 10, 11, 18]. As the previous studies have applied cultures or PCR-based methods, the results were quite incomparable with our data – in case of *S. pyogenes* we showed an incidence of 66 and 70% as opposed to the previous highest finding of 42% [19]. However, in nearly one third of our samples we did not detect *S. pyogenes*, strongly suggesting the existence of other causative agents of PTA in a significant proportion of patients. According to the current diagnostics practices, these other possible agents, especially anaerobic bacteria may remain frequently undetected.

***Fusobacterium necrophorum*** has been suggested as another possible causative agent of PTA [13, 20]. Studies applying NGS have detected *F. necrophorum* seldom in the healthy tonsils while both the prevalence and abundance of this species was significantly increased in case of tonsillitis [21]. In our study, we identified *F. necrophorum* in both sample types for over 75% of PTA patients with high abundancies in half of non-*S. pyogenes* cases, indicating a clear link between PTA development and *F. necrophorum*. Thus, our data are in line with other studies suggesting that *F. necrophorum* might be a substantial causative agent of PTA [3, 8, 20]. A synergistic effect of *F. necrophorum* and *S. pyogenes* in the formation of PTA is unlikely provided the observed negative correlation between the two species. *Streptococcus pyogenes* may have an inhibitory effect on *F. necrophorum* as described earlier [22]. Considering these complex relationships between the microbes, it is important to consider *F. necrophorum* as the causative agent of PTA in the absence of *S. pyogenes* and vice versa.

***Fusobacterium nucleatum*** ranked third in terms of mean relative abundance in both sample types and was the third most frequently predominating species in individual samples as well. It predominated in 15% of the communities, suggesting that it could belong to the main causative agents in those cases. This species has not previously been considered a potential cause of PTA, except in a patient with Lemierre syndrome [23]. At the same time, it has been isolated from PTA patients in several studies [24–26], including in our previous culture-based study [13]. In addition, significant increase in antibodies titre against *F. nucleatum* has been found previously in PTA patients [27]. Our results showed a significant increase in the abundance of *F. nucleatum* in the absence of *F. necrophorum* and a negative correlation between the two. Again, an antagonistic relationship is a plausible possibilty.

In addition to the above-mentioned three bacteria, or data suggest that some PTA cases may be associated with **other opportunistic bacteria** like some streptococcal species (*S. anginosus, S. mitis*) or other Gram negative anaerobic rods (*Porphyromonas, Prevotella*). Although *S. pyogenes* and *F. necrophorum* tended to cause the infection rather individually than in combination, this disease might be polymicrobial in case of other microbial combinations [10]. To clarify the clinical importance of other bacteria, additional studies are needed.

Correlations between different bacteria and clinical findings were mild in our study. We found *S. pyogenes* to be associated with increased duration of symptoms before hospitalization possibly indicating a slower development of severe symptoms as compared to fusobacteria.

The **sampling sites** for microbiological analyses need to be chosen wisely [17]. In our study we compared biopsy material from tonsils and pus. The tonsils displayed significantly higher levels of less pathogenic and environmental bacteria in terms of both incidence and relative abundances, such as *Chryseobacterium hominis* and *Sphingomonas faeni*, and also the poorly defined *Allorhizobium-Neorhizobium-Pararhizobium-Rhizobium* group. Although *C. hominis* is considered to be of human origin [28] and *S. faeni* an environmental microbe [29], a positive correlation was shown between them. Their role in the disease should be evaluated with caution, since contamination cannot be ruled out. Additionally, tonsils form an important part of localised immune protection and they are exposed to various microbes both inhaled and ingested [14]. This aspect may also explain the higher content of *S. faeni, C. hominis* and *Allorhizobium* group in the tonsil tissue as compared to pus. Specimen collection and handling took place in the same environment for pus and biopsies. As concerns putative causative agents of PTA, there was no significant difference between their prevalence in pus and tonsils. In clinical practice, pus has several advantages over tonsil biopsies: it is easier both to collect and to manage in the laboratory and it was found to be less contaminated by non-pathogenic bacteria complication interpretation of results. Therefore, we recommended aspiration of pus for routine diagnostic bacterial diagnostics. However, it is important to note that in case of cultures, microbes in pus may not always be viable.

**Treatment approaches** for PTA patients vary remarkably, depending on physicians’ preference and also regional aspects considering antibiotic susceptibility. The first choice of antibiotic for PTA is frequently penicillin alone or in combination with metronidazole. Other common antibiotic treatment options include amoxicillin in combination with metronidazole, cephalosporins or clindamycin [11, 30]. Penicillin is effective against *S. pyogenes* and in most cases targets fusobacteria as well. Recent evidence reports a continuous susceptible state and even a decrease in MIC values of common antibiotics used for fusobacterial infections, such as penicillin, clindamycin and others [31, 32]. Thus, penicillin remains an effective treatment option in majority of PTA cases. However, the possibility of other anaerobes cannot be ruled out, therefore, the addition of specific anaerobe coverage with metronidazole or clindamycin in poor response to penicillin alone should be considered. In addition, the polyols erythritol and xylitol inhibit growth of *S. pyogenes* and thus may have potential in preventing PTA in patients with frequent episodes of tonsillitis [33].

**Limitations.** Our study cohort was predominated by male patients. However, some studies have identified male gender as a risk factor of PTA [34], which could help to explain this phenomenon. Another limitation was antibiotics usage prior hospitalization that might influence microbial community composition by reducing the microbial diversity [17]. Antibiotics are often useful in order to treat the tonsillitis and to prevent several complications [3]. At the same time, culture independent methods are less prone to bias caused by antimicrobial treatment in particular in sample types with slow clearance of microbial DNA like undrained abscesses. To our knowledge, this is the first report using NGS, the new golden standard of microbiome studies, for identifying the microbial communities in tonsils and pus of PTA patients. Though being useful in revealing the wide spectrum of causative agents, it does not allow to determine their antibiotic susceptibility if needed.

**In conclusion**, the most probable causative agents of PTA include *Streptococcus pyogenes, Fusobacterium necrophorum* and *Fusobacterium nucleatum.* Some other streptococci (e.g. *S. anginosus*) and anaerobes (e.g. *Prevotella* and *Porphyromonas*) might have role in PTA development, too. Hence, it is important to consider anaerobic bacteria both in the laboratory work and the treatment of PTA. Pus from the peritonsillar abscess appears to be a more representative specimen for microbiological examination than the tonsillar tissue. Our results are important in the context of optimizing the handling of the PTA patients.

### Electronic supplementary material

Below is the link to the electronic supplementary material.

Supplementary Material 1: Fig. S1. NMDS cluster analysis of microbial diversity and possible confounders. Preliminary assessments from of NMDS cluster analysis did not reveal correlation between microbial diversity and smoking status (A), age (B) and antibiotic intake before hospitalization (C). Fig. S2. Relative distribution of streptococci (blue colors), anaerobic bacteria (green colors) and other bacteria (yellow-orange colors) in pus samples of each particular patient. Table S1. Details of molecular methods. Table S2 Incidence and abundance of phyla in pus and tonsils of PTA patients. Table S3. Incidence and abundance of classes in pus and tonsils of PTA patients. Table S4. Incidence and abundance of genera in pus and tonsils of PTA patients. Table S5. Incidence and abundance of species in pus and tonsils of PTA patients.

## Data Availability

All data generated or analysed during this study are included in this published article and its supplementary information files.

## References

[1] Menegas S, Moayedi S, Torres M (2021). Abscess Management: an evidence-based review for Emergency Medicine clinicians. J Emerg Med.

[2] Galioto NJ (2017). Peritonsillar Abscess. Am Fam Physician.

[3] Klug TE, Greve T, Hentze M (2020). Complications of peritonsillar abscess. Ann Clin Microbiol Antimicrob.

[4] Blair AB, Booth R, Baugh R (2015). A unifying theory of tonsillitis, intratonsillar abscess and peritonsillar abscess. Am J Otolaryngol.

[5] Kordeluk S, Novack L, Puterman M, Kraus M, Joshua BZ (2011). Relation between peritonsillar Infection and acute tonsillitis: myth or reality?. Otolaryngol Head Neck Surg.

[6] Vanhapiha N, Sanmark E, Blomgren K, Wiksten J (2022). Minor salivary gland Infection as origin of peritonsillitis - novel theory and preliminary results. Acta Otolaryngol.

[7] Hidaka H, Kuriyama S, Yano H, Tsuji I, Kobayashi T (2011). Precipitating factors in the pathogenesis of peritonsillar abscess and bacteriological significance of the Streptococcus milleri group. Eur J Clin Microbiol Infect Dis.

[8] Wiksten JE, Laakso S, Maki M, Makitie AA, Pitkaranta A, Blomgren K (2015). Microarray identification of bacterial species in peritonsillar abscesses. Eur J Clin Microbiol Infect Dis.

[9] Klug TE, Henriksen JJ, Rusan M, Fuursted K, Krogfelt KA, Ovesen T (2014). Antibody development to Fusobacterium necrophorum in patients with peritonsillar abscess. Eur J Clin Microbiol Infect Dis.

[10] Plum AW, Mortelliti AJ, Walsh RE (2015). Microbial Flora and Antibiotic Resistance in Peritonsillar abscesses in Upstate New York. Ann Otol Rhinol Laryngol.

[11] Slouka D, Hanakova J, Kostlivy T, Skopek P, Kubec V, Babuska V (2020). Epidemiological and microbiological aspects of the Peritonsillar Abscess. Int J Environ Res Public Health.

[12] Tsai YW, Liu YH, Su HH (2018). Bacteriology of peritonsillar abscess: the changing trend and predisposing factors. Braz J Otorhinolaryngol.

[13] Vaikjärv R, Kasenõmm P, Jaanimäe L, Kivisild A, Rööp T, Sepp E, Mändar R (2016). Microbiology of peritonsillar abscess in the South Estonian population. Microb Ecol Health Dis.

[14] Masters KG, Zezoff D, Lasrado S, Anatomy. Head and Neck, Tonsils. In: StatPearls. Treasure Island (FL)2022. 30969614

[15] Powell J, Wilson JA (2012). An evidence-based review of peritonsillar abscess. Clin Otolaryngol.

[16] Vaikjärv R, Mändar R, Kasenõmm P (2019). Peritonsillar abscess is frequently accompanied by sepsis symptoms. Eur Arch Otorhinolaryngol.

[17] Kumpitsch C, Koskinen K, Schopf V, Moissl-Eichinger C (2019). The microbiome of the upper respiratory tract in health and Disease. BMC Biol.

[18] Lepelletier D, Pinaud V, Le Conte P, Bourigault C, Asseray N, Ballereau F (2016). Peritonsillar abscess (PTA): clinical characteristics, microbiology, drug exposures and outcomes of a large multicenter cohort survey of 412 patients hospitalized in 13 French university hospitals. Eur J Clin Microbiol Infect Dis.

[19] Gavriel H, Lazarovitch T, Pomortsev A, Eviatar E (2009). Variations in the microbiology of peritonsillar abscess. Eur J Clin Microbiol Infect Dis.

[20] Ehlers Klug T, Rusan M, Fuursted K, Ovesen T (2009). Fusobacterium necrophorum: most prevalent pathogen in peritonsillar abscess in Denmark. Clin Infect Dis.

[21] Jensen A, Fagö-Olsen H, Sørensen CH, Kilian M (2013). Molecular Mapping to species Level of the Tonsillar Crypt Microbiota Associated with Health and recurrent Tonsillitis. PLoS ONE.

[22] Brook I, Walker RI (1986). The relationship between *Fusobacterium* species and other flora in mixed Infection. J Med Microbiol.

[23] Kumar D, Shamsi WE, Gomes T, Warsha F (2021). Forgotten Disease: an atypical case of Lemierre syndrome presenting as a soft tissue abscess. BMJ Case Rep.

[24] Brook I, Foote PA, Slots J (1995). Immune response to Fusobacterium nucleatum and Prevotella intermedia in patients with peritonsillar cellulitis and abscess. Clin Infect Dis.

[25] Albertz N, Nazar G (2012). Peritonsillar abscess: treatment with immediate tonsillectomy – 10 years of experience. Acta Otolaryngol.

[26] Jousimies-Somer H, Savolainen S, Mäkitie A, Ylikoski J (1993). Bacteriologic findings in peritonsillar abscesses in young adults. Clin Infect Dis.

[27] Brook I (1994). Fusobacterial Infections in children. J Infect.

[28] Vaneechoutte M, Kämpfer P, De Baere T, Avesani V, Janssens M, Wauters G. *Chryseobacterium hominis* sp. nov., to accomodate clinical isolates biochemically similar to CDC groups II-h and II-c. Int J Syst Evol MicroBiol. 2007;57(11):2623$$-$$2628. 10.1099/ijs.0.65158-0 17978230

[29] Busse H-J, Denner EBM, Buczolits S, Salkinoja-Salonen M, Bennasar A, Kämpfer P. *Sphingomonas aurantiaca* sp. nov., *Sphingomonas aerolata* sp. nov. and *Sphingomonas faeni* sp. nov., air- and dustborne and Antarctic, orangepigmented, psychrotolerant bacteria, and emended description of the genus *Sphingomonas* International Journal of Systematic and Evolutionary Microbiology. 2003; 53(5): 1253$$-$$1260. 10.1099/ijs.0.02461-0 13130003

[30] Wiksten J, Blomgren K, Eriksson T, Guldfred L, Bratt M, Pitkaranta A (2014). Variations in treatment of peritonsillar abscess in four nordic countries. Acta Otolaryngol.

[31] Lõivukene K, Kermes K, Sepp E, Naaber P, Mändar R, Kõljalg S (2023). Trends in the prevalence and antibiotic susceptibility of anaerobic gram-negative bacteria causing clinical Infections in Estonia. SLJID.

[32] Kim M, Yun SY, Lee Y, Lee H, Yong D, Lee K. Clinical Differences in Patients Infected with *Fusobacterium* and Antimicrobial Susceptibility of *Fusobacterium* isolates Recovered at a Tertiary-Care Hospital in Korea. Annals of Laboratory Medicine. 2022;42(2):188$$-$$195. 10.3343/alm.2022.42.2.188 PMC8548237 34635612

[33] Kõljalg S, Vaikjärv R, Smidt I, Rööp T, Chakrabarti A, Kasenõmm P, Mändar R (2021). Effect of erythritol and xylitol on *Streptococcus pyogenes* causing peritonsillar abscesses. Sci Rep.

[34] Matsuda A, Tanaka H, Kanaya T, Kamata K, Hasegawa M (2002). Peritonsillar abscess: a study of 724 cases in Japan. Ear Nose Throat J.

