# Supplementary material for: Unveiling the etiology of peritonsillar abscess using next generation sequencing
Source: Ann Clin Microbiol Antimicrob. 2023 Nov 8;22:98. doi: 10.1186/s12941-023-00649-0 (PMC10633907; doi:10.1186/s12941-023-00649-0)
Supplement: Supplementary file 1 — Supplementary Material 1: Fig. S1. NMDS cluster analysis of microbial diversity and possible confounders. Preliminary assessments from of NMDS cluster analysis did not reveal correlation between microbial diversity and smoking status (A), age (B) and antibiotic intake before hospitalization (C). Fig. S2. Relative distribution of streptococci (blue colors), anaerobic bacteria (green colors) and other bacteria (yellow-orange colors) in pus samples of each particular patient. Table S1. Details of molecular methods. Table S2 Incidence and abundance of phyla in pus and tonsils of PTA patients. Table S3. Incidence and abundance of classes in pus and tonsils of PTA patients. Table S4. Incidence and abundance of genera in pus and tonsils of PTA patients. Table S5. Incidence and abundance of species in pus and tonsils of PTA patients. [file 12941_2023_649_MOESM1_ESM.docx]

**Supplementary information**

| **A** | **B** |
| --- | --- |
| 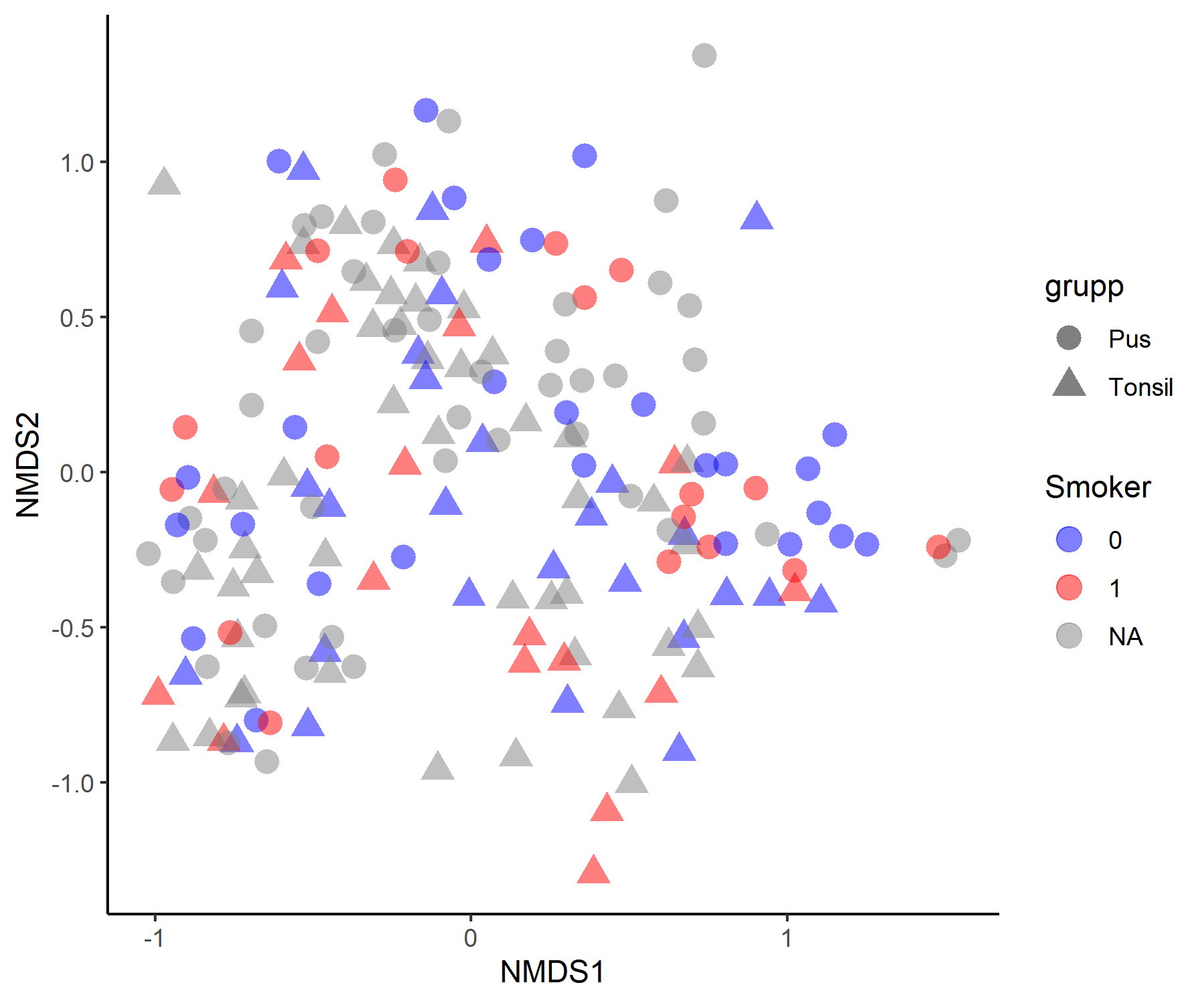 | 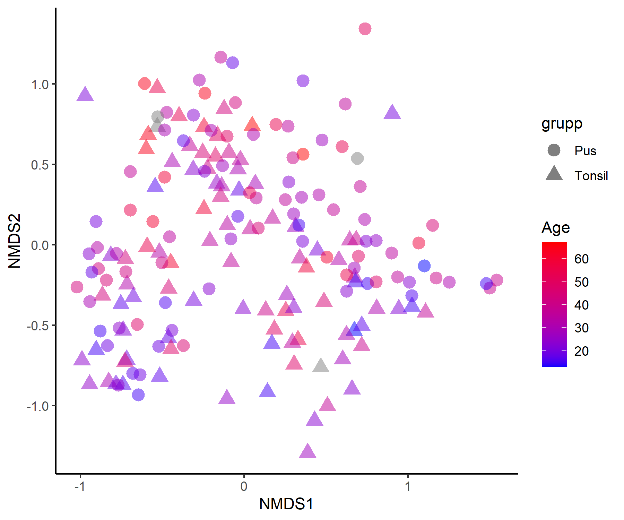 |
| **C** |  |
| 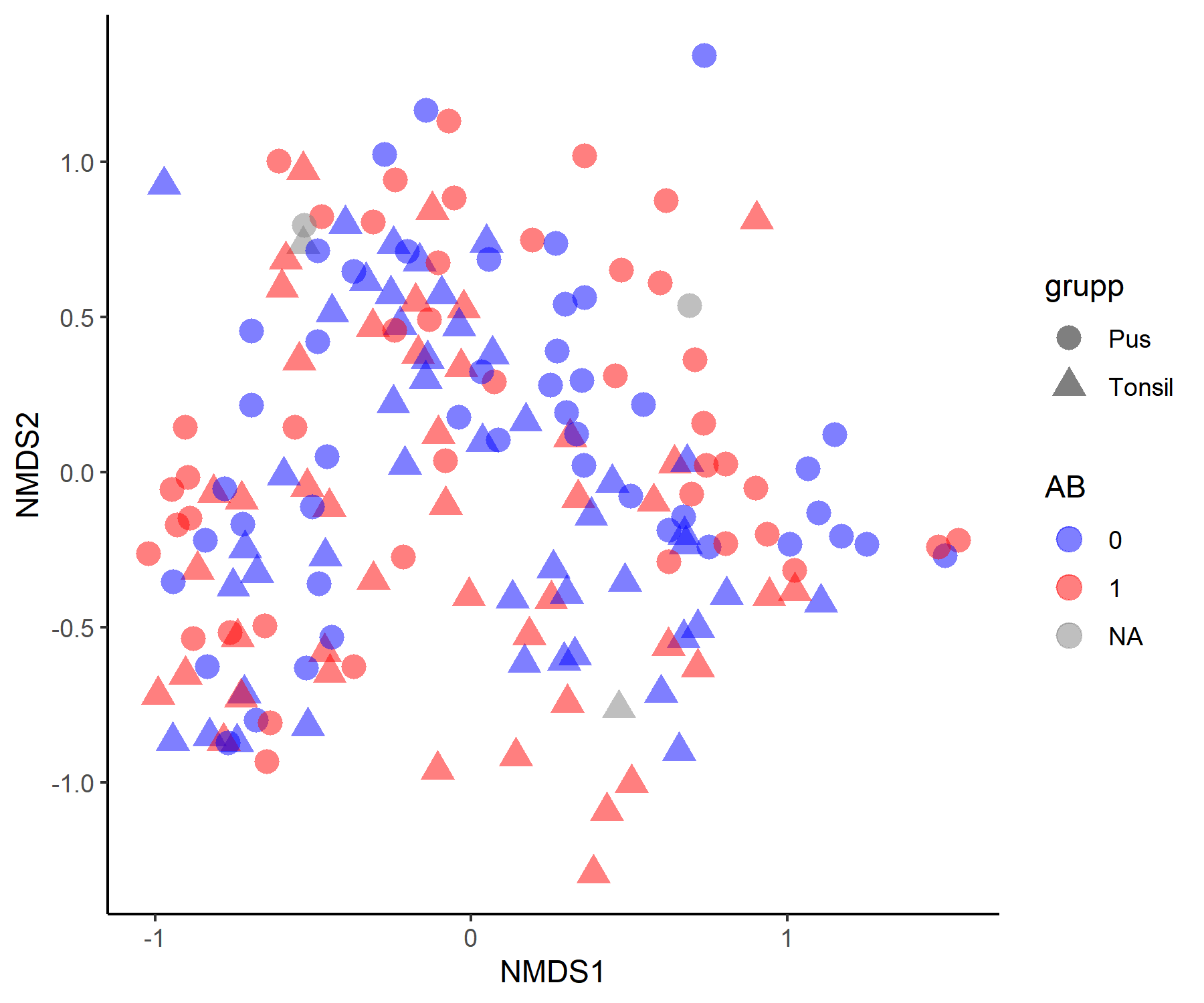 |  |

**Figure S1**. NMDS cluster analysis of microbial diversity and possible confounders. Preliminary assessments from of NMDS cluster analysis did not reveal correlation between microbial diversity and smoking status (A), age (B) and antibiotic intake before hospitalization (C).

**
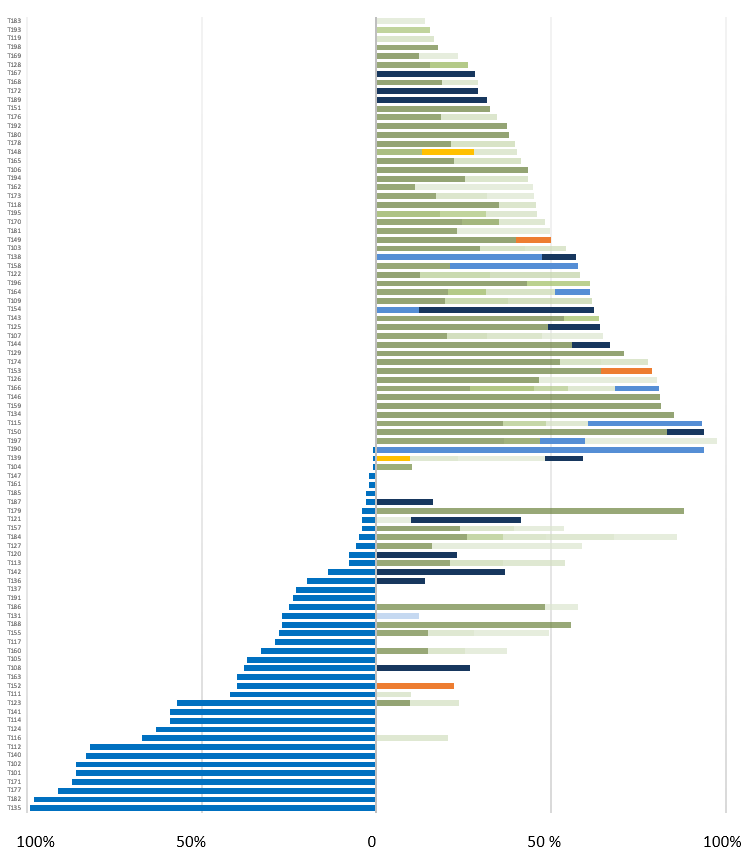
**

**
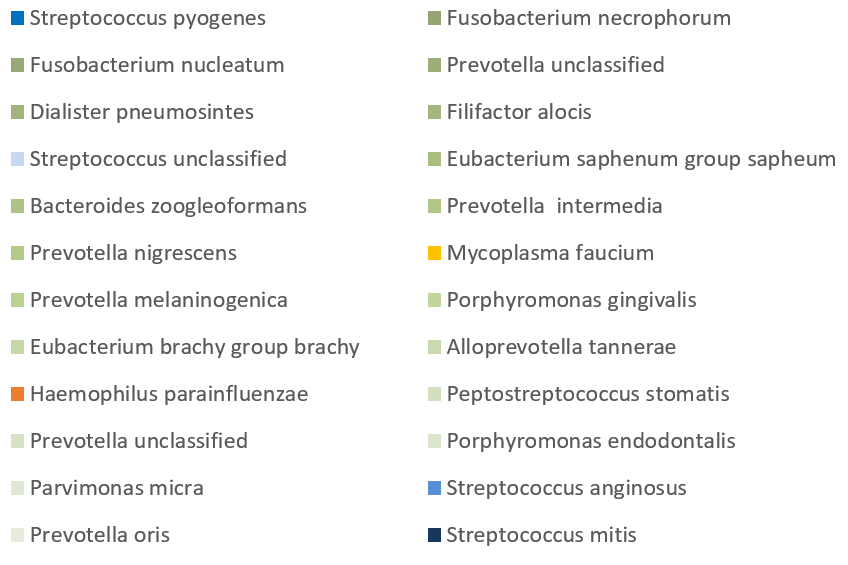
**

**Figure S2**. Relative distribution of streptococci (blue colors), anaerobic bacteria (green colors) and other bacteria (yellow-orange colors) in pus samples of each particular patient.

**Table S1**. Details of molecular methods

| **DNA extraction** |
| --- |
| Bacterial DNA from washouts of the swabs was extracted with the PureLinkTM Microbiome DNA Purification Kit (Invitrogen, USA), using a ELMI Sky Line instrument (ELMI Ltd, Riga, Latvia) according to manufacturer’s instructions.  The frozen tonsil biopsy specimens (~25 mg) were suspended in 500 µL of lysis buffer (200 mM Tris-HCl (pH 8.0), 25 mM ethylenediaminetetraacetic acid (EDTA), 300 mM NaCl, 1.2% sodium dodecyl sulfate) and 20 µL of proteinase K (400 µg/mL) for DNA extraction. The mixture was incubated at 37 ◦C for 24 h. The procedure of DNA extraction was continued according to the tissue protocol of QIAamp DNA Mini Kit (Qiagen, Hilden, Germany).  Extracted DNA samples were quantified with Qubit fluorometer (ThermoFisher Scientific, USA) and diluted to 5 ng/μl. |
| **Illumina sequencing** |
| DNA was amplified using primers:  16S_F (5′- TCGTCGGCAGCGTCAGATGTGTATAAGAGACAGCCTACGGGNGGCWGCAG -3′) and 16S_R (5′-GTCTCGTGGGCTCGGAGATGTGTATAAGAGACAGGACTACHVGGGTATCTAATCC-3′) for PCR amplification of an approximately 460 bp region within the hypervariable (V3-V4) region of prokaryotic 16S ribosomal RNA gene (Klindworth et al. 2013). The first PCR mixture contained 12.5 μL of KAPA HiFi HotStart ReadyMix (2X) (Kapa Biosystems, Wilmington, MA), 1 μL of each primer (10 μM) and 6 μL of template DNA (5 ng/μL). The reaction volume was brought to 25 μL with milliQ water. PCR conditions were 95 °C for 3 min followed by 24 cycles of 95 °C for 30 sec, 55 °C for 30 sec, and 72 °C for 30 sec with a final extension at 72 °C for 5 min. The PCR products were purified using a 0.8x solution of AMPure XP Beads (Beckman Coulter, Inc.). The purified products were quantified with Qubit, diluted to 10 ng/μL and used as a template for indexing PCR. Indexes and sequencing adapters were attached to the PCR products in the indexing PCR, using Illumina Nextera XT dual index primers (Illumina Inc., San Diego, CA). The indexing PCR contained 5 μL of each index primer, 15 μL of KAPA HiFi HotStart ReadyMix (2X) and 5 μL of template DNA (10 ng/μL).  The second PCR cycling conditions were 95 °C for 3 min followed by 7 cycles of 95 °C for 30 sec, 55 °C for 30 sec, and 72 °C for 30 sec with a final extension at 72 °C for 5 min. Indexed PCR products were purified using a 1.9x solution of AMPure XP Beads, quantified and combined into a final library pool in equimolar concentrations. The library pool was quantified using Illumina-specific KAPA Library Quant Kit (Kapa Biosystems, Wilmington, MA). Sequencing was carried out on an Illumina MiSeq System using MiSeq Reagent Kit v3 in paired end 2 × 300 bp mode.  DNA sequence data was analysed using BION-meta, currently unpublished open source programme, according to authors’ instructions (McDonald et al., 2016). First, sequences were cleaned at both ends using a 99.5% minimum quality threshold for at least 18 of 20 bases for 50 -end and 28 of 30 bases for 30 -end, then joined, followed by removal of contigs shorter than 350 bp. The sequences were cleaned from chimaeras and clustered by 95% oligonucleotide similarity (kimer length of 8 bp, step size 2 bp). Lastly, consensus reads were aligned to the SILVA reference 16S rDNA database (v123) using word length of 8 and similarity cut-off of 90. |
| **References** |
| Klindworth, A., Pruesse, E., Schweer, T., Peplies, J., Quast, C., Horn, M., Glöckner, F. O. (2013). Evaluation of general 16S ribosomal RNA gene PCR primers for classical and next-generation sequencing-based diversity studies. *Nucleic Acids Research,* 41(1): e1.  McDonald, J. E., Larsen, N., Pennington, A., Connolly, J., Wallis, C., Rooks, D. J., Hall, N., McCarthy, A. J., Allison, H. E. (2016). Characterising the Canine Oral Microbiome by Direct Sequencing of Reverse-Transcribed rRNA Molecules. *PLoS One,* 11(6): e0157046.  Quast, C., Pruesse, E., Yilmaz, P., Gerken, J., Schweer, T., Yarza, P., Peplies, J., Glöckner, F. O. (2013). The SILVA ribosomal RNA gene database project: improved data processing and web-based tools. *Nucleic Acids Research,* 41(D1): D590−D296 |

**Table S2.** Incidence and abundance of phyla in pus and tonsils of PTA patients

| **Phylum** | | **Tonsils** | | **Pus** | | ***P* for incidence** | ***P* for abundance** |
| --- | --- | --- | --- | --- | --- | --- | --- |
|  |  | % positive | Mean ± SD | % positive | Mean ± SD |  |  |
|  |  |  | Median (Q1; Q3) |  | Median (Q1; Q3) |  |  |
| *Firmicutes* | | 100,00 | 28,22±20,23 | 100,00 | 44,78±24,69 | NS* | <0,001 |
|  |  |  | 24,32 (13,43; 35,21) |  | 38,69  (26,92; 57,23) |  |  |
| *Bacteroidota* | | 100,00 | 26,16±16,13 | 100,00 | 21,07±14,54 | NS | 0,04 |
|  |  |  | 25,31  (12,90; 38,40) |  | 23,55  (6,83; 33,76) |  |  |
| *Proteobacteria* | | 100,00 | 16,92±19,39 | 100,00 | 6,16±7,52 | NS | <0,001 |
|  |  |  | 8,94  (4,45; 19,21) |  | 3,80  (1,26;7,88) |  |  |
| *Fusobacteriota* | | 100,00 | 23,44±24,95 | 98,90 | 22,91±23,02 | NS | NS |
|  |  |  | 12,82  (5,89; 28,47) |  | 15,43  (4,60; 35,79) |  |  |
| *Actinobacteriota* | | 100,00 | 2,04±4,02 | 98,90 | 2,96±3,20 | NS | 0,02 |
|  |  |  | 1,07  (0,56;2,25) |  | 1,64  (0,69; 4,27) |  |  |
| *Spirochaetota* | | 76,92 | 1,77±2,78 | 78,02 | 1,19±2,33 | NS | NS |
|  |  |  | 0,36  (0,04; 2,40) |  | 0,18  (0,03; 1,08) |  |  |
| *Patescibacteria* | | 67,03 | 0,36±0,71 | 78,02 | 0,37±0,69 | NS | NS |
|  |  |  | 0,08  (0; 0,43) |  | 0,10  (0,02; 0,32) |  |  |
| *Cyanobacteria* | | 59,34 | 0,08±0,10 | 47,25 | 0,11±0,53 | NS | 0,05 |
|  |  |  | 0,02  (0; 0,12) |  | 0  (0;0,06) |  |  |
| *Synergistota* | | 50,55 | 0,66±1,63 | 48,35 | 0,09±0,23 | NS | NS |
|  |  |  | 0,02  (0; 0,33) |  | 0  (0; 0,05) |  |  |
| *Gracilibacteria* | 25,27 | 0,13±0,48 | 30,77 | 0,06±0,025 | NS | NS |  |
|  |  | 0  (0; 0,01) |  | 0  (0; 0,02) |  |  |  |
| *Desulfobacterota* | 25,27 | 0,06±0,22 | 10,99 | 0,01±0,02 | NS | 0,01 |  |
|  |  | 0  (0; 0,01) |  | 0  (0; 0) |  |  |  |
| *Verrucomicrobiota* | 24,18 | 0,01±0,03 | 13,19 | 0,01±0,02 | NS | NS |  |
|  |  | 0  (0; 0) |  | 0  (0; 0) |  |  |  |
| *Chloroflexi* | 14,29 | 0,03±0,11 | 8,79 | 0±0,02 | NS | NS |  |
|  |  | 0  (0; 0) |  | 0  (0; 0) |  |  |  |
| *Euryarchaeota* | 8,79 | 0,03±0,20 | 6,59 | 0±0,01 | NS | NS |  |
|  |  | 0  (0; 0) |  | 0  (0; 0) |  |  |  |
| *Acidobacteriota* | 6,59 | 0±0,01 | 5,49 | 0,01±0,05 | NS | NS |  |
|  |  | 0  (0; 0) |  | 0  (0; 0) |  |  |  |
| *Deinococcota* | 3,30 | 0±0,01 | 3,30 | 0±0,01 | NS | NS |  |
|  |  | 0  (0; 0) |  | 0  (0; 0) |  |  |  |
| *Planctomycetota* | 2,20 | 0±0 | 2,20 | 0±001 | NS | NS |  |
|  |  | 0  (0; 0) |  | 0  (0; 0) |  |  |  |
| *Myxococcota* | 1,10 | 0±0 | 3,30 | 0±0,01 | NS | NS |  |
|  |  | 0  (0; 0) |  | 0  (0; 0) |  |  |  |
| *Parabasalia* | 1,10 | 0±0 | 2,20 | 0±0 | NS | NS |  |
|  |  | 0  (0; 0) |  | 0  (0; 0) |  |  |  |
| *Gemmatimonadota* | 1,10 | 0±0,02 | 1,10 | 0±0,01 | NS | NS |  |
|  |  | 0  (0; 0) |  | 0  (0; 0) |  |  |  |
| *Elusimicrobiota* | 1,10 | 0±0,01 | 1,10 | 0±0 | NS | NS |  |
|  |  | 0  (0; 0) |  | 0  (0; 0) |  |  |  |
| *Thermoplasmatota* | 1,10 | 0±0 | 0 | 0±0 | NS | NS |  |
|  |  | 0  (0; 0) |  | 0  (0; 0) |  |  |  |
| *Bdellovibrinota* | 0 | 0±0 | 5,49 | 0±0 | NS | 0,02 |  |
|  |  | 0  (0;0) |  | 0  (0; 0) |  |  |  |
| *Parcubacteria* | 0 | 0±0 | 3,30 | 0±0,01 | NS | NS |  |
|  |  | 0  (0; 0) |  | 0  (0; 0) |  |  |  |
| *Candidatus Falkowbacteria* | 0 | 0±0 | 1,10 | 0±0 | NS | NS |  |
|  |  | 0  (0;0) |  | 0  (0; 0) |  |  |  |
| *Candidatus Lloydbacteria* | 0 | 0±0 | 1,10 | 0±0 | NS | NS |  |
|  |  | 0  (0;0) |  | 0  (0; 0) |  |  |  |
| *Candidatus Zambryskibacteria* | 0 | 0±0 | 1,10 | 0±0 | NS | NS |  |
|  |  | 0  (0;0) |  | 0  (0; 0) |  |  |  |
| *Cercozoa* | 0 | 0±0 | 1,10 | 0±0 | NS | NS |  |
|  |  | 0  (0;0) |  | 0  (0; 0) |  |  |  |
| *Cloacimonadota* | 0 | 0±0 | 1,10 | 0±0 | NS | NS |  |
|  |  | 0  (0;0) |  | 0  (0; 0) |  |  |  |
| *Dependentiae* | 0 | 0±0 | 1,10 | 0±0 | NS | NS |  |
|  |  | 0  (0;0) |  | 0  (0; 0) |  |  |  |
| *Fibrobacterota* | 0 | 0±0 | 1,10 | 0±0 | NS | NS |  |
|  |  | 0  (0;0) |  | 0  (0; 0) |  |  |  |
| *Methylomirabilota* | 0 | 0±0 | 1,10 | 0±0 | NS | NS |  |
|  |  | 0  (0;0) |  | 0  (0; 0) |  |  |  |
| *Nitrospirota* | 0 | 0±0 | 1,10 | 0±0 | NS | NS |  |
|  |  | 0  (0;0) |  | 0  (0; 0) |  |  |  |

*NS – not significant

**Table S3**. Incidence and abundance of classes in pus and tonsils of PTA patients

| **Phylum** | **Class** | **Tonsils** | | | | **Pus** | | ***P* for incidence** | ***P* for abundance** | |
| --- | --- | --- | --- | --- | --- | --- | --- | --- | --- | --- |
|  |  | % positive | Mean±SD | | | % positive | Mean±SD |  |  |  |
|  |  |  | Median  (Q1; Q3) | | |  | Median  (Q1; Q3) |  |  |  |
| *Firmicutes* | *Bacilli* | 100,00 | 17,24±20,42 | | | 100,00 | 31,02±28,36 | NS* | <0,001 | |
|  |  |  | 9,13  (3,73; 22,25) | | |  | 18,77  (8,19; 48,57) |  |  |  |
|  | *Clostridia* | 100,00 | 6,16±4,96 | | | 98,90 | 9,89±10,09 | NS | NS | |
|  |  |  | 4,77  (1,63; 9,86) | | |  | 5,42  (1,96;14,97) |  |  |  |
|  | *Negativicutes* | 100,00 | 4,81±4,46 | | | 98,90 | 3,87±3,47 | NS | NS | |
|  |  |  | 3,59  (1,65; 6,55) | | |  | 3,10  (1,20; 5,35) |  |  |  |
|  | *Syntrophomonadia* | 3,30 | 0,01±0,05 | | | 0 | 0±0 | NS | NS | |
|  |  |  | 0  (0; 0) | | |  | 0  (0; 0) |  |  |  |
|  | *Thermoanaerobacteria* | 1,10 | 0±0,02 | | | 0 | 0±0 | NS | NS | |
|  |  |  | 0  (0; 0) | | |  | 0  (0; 0) |  |  |  |
|  | *Limnochordia* | 0 | 0±0 | | | 1,10 | 0±0 | NS | NS | |
|  |  |  | 0  (0; 0) | | |  | 0  (0; 0) |  |  |  |
| *Bacteroidota* | *Bacteroidia* | 100,00 | 26,16±16,13 | | | 100,00 | 21,07±14,54 | NS | 0,04 | |
|  |  |  | 25,31  (12,90; 38,40) | | |  | 23,55  (6,83; 33,76) |  |  |  |
| *Proteobacteria* | *Alphaproteobacteria* | 100,00 | 11,20±15,81 | | | 70,33 | 0,50±1,21 | <0,001 | <0,001 | |
|  |  |  | 4,79  (1,43; 12,11) | | |  | 0,04  (0; 0,32) |  |  |  |
|  | *Gammaproteobacteria* | 98,90 | 4,82±10,07 | | | 97,80 | 4,97±7,21 | NS | NS | |
|  |  |  | 1,64  (0,81; 4,07) | | |  | 1,79  (0,46; 6,57) |  |  |  |
|  | *Campylobacterota* | 80,22 | 0,91±1,84 | | | 89,01 | 0,69±1,04 | NS | NS | |
|  |  |  | 0,44  (0,07; 0,84) | | |  | 0,31  (0,06; 0,76) |  |  |  |
|  | *Oligoflexia* | 0 | 0±0 | | | 2,20 | 0±0 | NS | NS | |
|  |  |  | 0  (0; 0) | | |  | 0  (0; 0) |  |  |  |
| *Fusobacteriota* | *Fusobacteriia* | 100,00 | 23,44±24,95 | | | 98,90 | 22,91±23,02 | NS | NS | |
|  |  |  | 12,82  (5,89; 28,47) | | |  | 15,43  (4,60; 35,79) |  |  |  |
| *Actinobacteriota* | *Actinobacteriia* | 98,90 | 1,53±3,79 | | | 97,80 | 2,22±2,40 | NS | 0,03 | |
|  |  |  | 0,67  (0,35; 1,65) | | |  | 1,31  (0,34; 3,40) |  |  |  |
|  | *Coriobacteriia* | 78,02 | 0,51±1,32 | | | 90,11 | 0,74±1,68 | NS | NS | |
|  |  |  | 0,20  (0,03; 0,43) | | |  | 0,19  (0,05; 0,60) |  |  |  |
|  | *Acidimicrobiia* | 0 | 0±0 | | | 6,59 | 0±0,01 | NS | 0,01 | |
|  |  |  | 0  (0; 0) | | |  | 0  (0; 0) |  |  |  |
|  | *Thermoleophilia* | 0 | 0±0 | | | 3,30 | 0±0,01 | NS | NS | |
|  |  |  | 0  (0; 0) | | |  | 0  (0; 0) |  |  |  |
| *Spirochaetota* | *Spirochaetia* | 76,92 | 1,77±2,78 | | | 78,02 | 1,19±2,33 | NS | NS | |
|  |  |  | 0,36  (0,04; 2,40) | | |  | 0,18  (0,03; 1,08) |  |  |  |
| *Patescibacteria* | *Saccharimonadia* | 67,03 | 0,36±0,71 | | | 78,02 | 0,37±0,69 | NS | NS | |
|  |  |  | 0,08  (0; 0,43) | | |  | 0,10  (0,02; 0,32) |  |  |  |
|  | *ABY1* | 0 | 0±0 | | | 1,10 | 0±0 | NS | NS | |
|  |  |  | 0  (0;0) | | |  | 0  (0; 0) |  |  |  |
| *Cyanobacteria* | *Cyanobacteriia* | 54,95 | 0,07±0,10 | | | 46,15 | 0,11±0,53 | NS | NS | |
|  |  |  | 0,02  (0; 0,11) | | |  | 0  (0; 0,06) |  |  |  |
|  | *Vampirivibrionia* | 13,19 | 0,01±0,02 | | | 4,40 | 0±0,02 | NS | 0,03 | |
|  |  |  | 0  (0; 0) | | |  | 0  (0; 0) |  |  |  |
| *Synergistota* | *Synergistia* | 50,55 | 0,66±1,63 | | | 48,35 | 0,09±0,23 | NS | NS | |
|  |  |  | 0,02  (0; 0,33) | | |  | 0  (0; 0,05) |  |  |  |
| *Gracilibacteria* | *Gracilibacteria* | 25,27 | 0,13±0,48 | 30,77 | | | 0,06±0,25 | NS | | NS |
|  |  |  | 0  (0;0,01) |  |  |  | 0  (0; 0,02) |  |  |  |
| *Desulfobacterota* | *Desulfovibrioia* | 17,58 | 0,05±0,21 | 5,49 | | | 0±0,02 | NS | | 0,01 |
|  |  |  | 0  (0; 0) |  |  |  | 0  (0; 0) |  |  |  |
|  | *Desulfobulbia* | 9,89 | 0,01±0,07 | 4,40 | | | 0±0,02 | NS | | NS |
|  |  |  | 0  (0; 0) |  |  |  | 0  (0; 0) |  |  |  |
|  | *Desulfuromonadia* | 0 | 0±0 | 1,10 | | | 0±0 | NS | | NS |
|  |  |  | 0  (0; 0) |  |  |  | 0  (0; 0) |  |  |  |
|  | *Syntrophia* | 0 | 0±0 | 1,10 | | | 0±0 | NS | | NS |
|  |  |  | 0  (0; 0) |  |  |  | 0  (0; 0) |  |  |  |
| *Verrucomicrobiota* | *Verrucomicrobiae* | 20,88 | 0,01±0,03 | 9,89 | | | 0±0,02 | NS | | 0,04 |
|  |  |  | 0  (0; 0) |  |  |  | 0  (0; 0) |  |  |  |
|  | *Lentisphaeria* | 3,30 | 0±0,01 | 1,10 | | | 0±0 | NS | | NS |
|  |  |  | 0  (0; 0) |  |  |  | 0  (0; 0) |  |  |  |
|  | *Omnitrophia* | 0 | 0±0 | 3,30 | | | 0±0 | NS | | NS |
|  |  |  | 0  (0; 0) |  |  |  | 0  (0; 0) |  |  |  |
| *Chloroflexi* | *Anaerolinae* | 12,09 | 0,03±0,11 | 4,40 | | | 0±0,01 | NS | | 0,05 |
|  |  |  | 0  (0; 0) |  |  |  | 0  (0; 0) |  |  |  |
|  | *Chloroflexia* | 1,10 | 0±0,01 | 2,20 | | | 0±0,01 | NS | | NS |
|  |  |  | 0  (0; 0) |  |  |  | 0  (0; 0) |  |  |  |
|  | *KD4-96* | 1,10 | 0±0 | 2,20 | | | 0±0 | NS | | NS |
|  |  |  | 0  (0; 0) |  |  |  | 0  (0; 0) |  |  |  |
|  | *Ktedonobacteria* | 1,10 | 0±0 | 0 | | | 0±0 | NS | | NS |
|  |  |  | 0  (0; 0) |  |  |  | 0  (0; 0) |  |  |  |
|  | *AD3* | 0 | 0±0 | | 1,10 | | 0±0 | NS | | NS |
|  |  |  | 0  (0; 0) | |  |  | 0  (0; 0) |  |  |  |
|  | *TK10* | 0 | 0±0 | | 1,10 | | 0±0 | NS | | NS |
|  |  |  | 0  (0; 0) | |  |  | 0  (0; 0) |  |  |  |
| *Euryarchaeota* | *Methanobacteria* | 8,79 | 0,03±0,20 | | 6,59 | | 0±0,01 | NS | | NS |
|  |  |  | 0  (0; 0) | |  |  | 0  (0; 0)8. |  |  |  |
| *Acidobacteriota* | *Acidobacteriae* | 5,49 | 0±0,01 | | 2,20 | | 0±0,03 | NS | | NS |
|  |  |  | 0  (0; 0) | |  |  | 0  (0; 0) |  |  |  |
|  | *Vicinamibacteria* | 1,10 | 0±0 | | 5,49 | | 0±0,01 | NS | | NS |
|  |  |  | 0  (0; 0) | |  |  | 0  (0; 0) |  |  |  |
|  | *Blastocatellia* | 1,10 | 0±0 | | 0 | | 0±0 | NS | | NS |
|  |  |  | 0  (0; 0) | |  |  | 0  (0; 0) |  |  |  |
| *Deinococcota* | *Deinococci* | 3,30 | 0±0,01 | | 3,30 | | 0±0,01 | NS | | NS |
|  |  |  | 0  (0; 0) | |  |  | 0  (0; 0) |  |  |  |
| *Planctomycetota* | *Planctomycetes* | 2,20 | 0±0 | | 2,20 | | 0±0,01 | NS | | NS |
|  |  |  | 0  (0; 0) | |  |  | 0  (0; 0) |  |  |  |
|  | *Phycisphaerae* | 0 | 0±0 | | 1,10 | | 0±0 | NS | | NS |
|  |  |  | 0  (0; 0) | |  |  | 0  (0; 0) |  |  |  |
| *Myxococcota* | *Polyangia* | 1,10 | 0±0 | | 3,30 | | 0±0,01 | NS | | NS |
|  |  |  | 0  (0; 0) | |  |  | 0  (0; 0) |  |  |  |
|  | *Myxococcia* | 0 | 0±0 | | 1,10 | | 0±0 | NS | | NS |
|  |  |  | 0  (0; 0) | |  |  | 0  (0; 0) |  |  |  |
| *Parabasalia* | *Trichomonadea* | 1,10 | 0±0 | | 2,20 | | 0±0 | NS | | NS |
|  |  |  | 0  (0; 0) | |  |  | 0  (0; 0) |  |  |  |
| *Gemmatimonadota* | *Longimicrobia* | 1,10 | 0±0,02 | | 0 | | 0±0,02 | NS | | NS |
|  |  |  | 0  (0; 0) | |  |  | 0  (0; 0) |  |  |  |
|  | *Gemmatimonadodetes* | 0 | 0±0 | | 1,10 | | 0±0 | NS | | NS |
|  |  |  | 0  (0; 0) | |  |  | 0  (0; 0) |  |  |  |
| *Elusimicrobiota* | *Lineage-IIa* | 1,10 | 0±0,01 | | 1,10 | | 0±0 | NS | | NS |
|  |  |  | 0  (0; 0) | |  |  | 0  (0; 0) |  |  |  |
| *Thermoplasmatota* | *Thermoplasmata* | 1,10 | 0±0 | | 0 | | 0±0 | NS | | NS |
|  |  |  | 0  (0; 0) | |  |  | 0  (0; 0) |  |  |  |
| *Bdellovibrinota* | *Bdellovibrionia* | 0 | 0±0 | | 3,30 | | 0±0 | NS | | NS |
|  |  |  | 0  (0; 0) | |  |  | 0  (0; 0) |  |  |  |
| *Parcubacteria* | *Parcubacteria* | 0 | 0±0 | | 5,49 | | 0±0,01 | NS | | 0,02 |
|  |  |  | 0  (0; 0) | |  |  | 0  (0; 0) |  |  |  |
| *Cloacimonadota* | *Cloacimonadia* | 0 | 0±0 | | 1,10 | | 0±0 | NS | | NS |
|  |  |  | 0  (0; 0) | |  |  | 0  (0; 0) |  |  |  |
| *Dependentiae* | *Babeliae* | 0 | 0±0 | | 1,10 | | 0±0 | NS | | NS |
|  |  |  | 0  (0; 0) | |  |  | 0  (0; 0) |  |  |  |
| *Fibrobacterota* | *Fibrobacteria* | 0 | 0±0 | | 1,10 | | 0±0 | NS | | NS |
|  |  |  | 0  (0; 0) | |  |  | 0  (0; 0) |  |  |  |
| *Methylomirabilota* | *Methylomirabilia* | 0 | 0±0 | | 1,10 | | 0±0 | NS | | NS |
|  |  |  | 0  (0; 0) | |  |  | 0  (0; 0) |  |  |  |
| *Nitrospirota* | *4-29-1* | 0 | 0±0 | | 1,10 | | 0±0 | NS | | NS |
|  |  |  | 0  (0; 0) | |  |  | 0  (0; 0) |  |  |  |

*NS – not significant

**Table S4**. Incidence and abundance of genera in pus and tonsils of PTA patients**

| **Phylum** | **Class** | **Genus** *** | **Tonsils** | | **Pus** | | ***P* for incidence** | ***P* for abundance** |
| --- | --- | --- | --- | --- | --- | --- | --- | --- |
|  |  |  | % positive | Mean ± SD | % positive | Mean ± SD |  |  |
|  |  |  |  | Median (Q1; Q3) |  | Median (Q1; Q3) |  |  |
| *Firmicutes* | *Bacilli* | *Streptococcus* | 98,9 | 14,32 ± 20,51 | 100 | 27,63 ± 28,93 | NS* | <0,001 |
|  |  |  |  | 5,07  (1,63; 16,37) |  | 13,5  (4,25; 40,76) |  |  |
|  |  | *Gemella* | 78,02 | 0,51 ± 0,86 | 89,01 | 0,92 ± 1,27 | NS | 0,02 |
|  |  |  |  | 0,19  (0,03; 0,59) |  | 0,45  (0,04; 1,35) |  |  |
|  |  | *Granulicatella* | 70,33 | 0,45 ± 0,77 | 82,42 | 0,57 ± 0,95 | NS | NS |
|  |  |  |  | 0,11  (0; 0,51) |  | 0,19  (0,05; 0,65) |  |  |
|  |  | *Bacillus* | 69,23 | 0,12 ± 0,27 | 34,07 | 0,02 ± 0,04 | <0,001 | <0,001 |
|  |  |  |  | 0,04  (0; 0,14) |  | 0  (0; 0,02) |  |  |
|  |  | *Mycoplasma* | 51,65 | 0,98 ± 3,74 | 57,14 | 0,65 ± 2,13 | NS | NS |
|  |  |  |  | 0,02  (0; 0,32) |  | 0,02  (0; 0,21) |  |  |
|  |  | *Solobacterium* | 52,75 | 0,24 ± 0,52 | 61,54 | 0,22 ± 0,56 | NS | NS |
|  |  |  |  | 0,02  (0; 0,22) |  | 0,02  (0; 0,12) |  |  |
|  |  | *Bulleidia* | 36,26 | 0,21 ± 0,5 | 54,95 | 0,43 ± 0,99 | <0,001 | 0,03 |
|  |  |  |  | 0  (0; 0,12) |  | 0,02  (0; 0,27) |  |  |
|  |  | *Lactobacillus* | 13,19 | 0,02 ± 0,12 | 51,65 | 0,09 ± 0,18 | <0,001 | <0,001 |
|  |  |  |  | 0  (0; 0) |  | 0,01  (0; 0,08) |  |  |
|  |  | *Staphylococcus* | 27,47 | 0,06 ± 0,16 | 53,85 | 0,16 ± 0,47 | <0,001 | <0,001 |
|  |  |  |  | 0  (0; 0,01) |  | 0,01  (0; 0,11) |  |  |
|  | *Clostridia* | *Parvimonas* | 85,71 | 1,93 ± 2,7 | 90,11 | 3,46 ± 4,95 | NS | NS |
|  |  |  |  | 1,01  (0,08; 2,60) |  | 1,17  (0,08; 5,21) |  |  |
|  |  | *Peptostreptococcus* | 72,53 | 0,63 ± 1,11 | 76,92 | 1,66 ± 4,12 | NS | NS |
|  |  |  |  | 0,20  (0; 0,76) |  | 0,12  (0,01; 0,98) |  |  |
|  |  | *Oribacterium* | 78,02 | 0,47 ± 0,83 | 73,63 | 0,76 ± 1,52 | NS | NS |
|  |  |  |  | 0,11  (0,02; 0,53) |  | 0,06  (0; 0,7) |  |  |
|  |  | *Filifactor* | 60,44 | 0,6 ± 0,85 | 62,64 | 0,59 ± 1,38 | NS | NS |
|  |  |  |  | 0,08  (0; 0,92) |  | 0,05  (0; 0,5) |  |  |
|  |  | *Catonella* | 64,84 | 0,32 ± 0,66 | 62,64 | 0,31 ± 0,71 | NS | NS |
|  |  |  |  | 0,07  (0; 0,34) |  | 0,05  (0; 0,25) |  |  |
|  |  | *Eubacterium nodatum group* | 56,04 | 0,22 ± 0,43 | 63,74 | 0,22 ± 0,49 | NS | NS |
|  |  |  |  | 0,05  (0; 0,25) |  | 0,03  (0; 0,21) |  |  |
|  |  | *Eubacterium* | 52,75 | 0,39 ± 0,81 | 75,82 | 1,16 ± 2,38 | <0,001 | 0,01 |
|  |  |  |  | 0,02  (0; 0,37) |  | 0,09  (0,01; 0,94) |  |  |
|  |  | *Lachnoanaerobaculum* | 49,45 | 0,1 ± 0,23 | 54,95 | 0,11 ± 0,26 | NS | NS |
|  |  |  |  | 0  (0; 0,09) |  | 0,02  (0; 0,08) |  |  |
|  |  | *Stomatobaculum* | 45,05 | 0,08 ± 0,15 | 53,85 | 0,11 ± 0,28 | NS | NS |
|  |  |  |  | 0 (0; 0,11) |  | 0,02  (0; 0,06) |  |  |
|  |  | *Mogibacterium* | 35,16 | 0,03 ± 0,08 | 51,65 | 0,08 ± 0,18 | 0,036 | 0,02 |
|  |  |  |  | 0 (0; 0,04) |  | 0,01  (0; 0,08) |  |  |
|  | *Negativicutes* | *Veillonella* | 94,51 | 2,69 ± 3,78 | 91,21 | 2 ± 2,5 | NS | NS |
|  |  |  |  | 1,11  (0,2; 3,58) |  | 0,87  (0,15; 3,37) |  |  |
|  |  | *Dialister* | 76,92 | 0,96 ± 1,59 | 82,42 | 1,14 ± 1,71 | NS | NS |
|  |  |  |  | 0,39  (0,02; 1,41) |  | 0,18  (0,03; 1,7) |  |  |
|  |  | *Selenomonas* | 78,02 | 0,61 ± 0,91 | 70,33 | 0,37 ± 0,82 | NS | 0,004 |
|  |  |  |  | 0,21  (0,02; 0,81) |  | 0,07  (0; 0,27) |  |  |
|  |  | *Megasphaera* | 56,04 | 0,3 ± 0,59 | 57,14 | 0,18 ± 0,43 | NS | NS |
|  |  |  |  | 0,05  (0; 0,33) |  | 0,02  (0; 0,12) |  |  |
| *Bacteroidota* | *Bacteroidia* | *Prevotella* | 100,00 | 13,42 ± 11,13 | 97,8 | 13,14 ± 10,63 | NS | NS |
|  |  |  |  | 11,48  (2,86; 20,78) |  | 11,77  (3,18; 19,63) |  |  |
|  |  | *Chryseobacterium* | 96,70 | 2,96 ± 4,2 | 38,46 | 0,06 ± 0,27 | <0,001 | <0,001 |
|  |  |  |  | 1,26  (0,42; 3,17) |  | 0  (0; 0,02) |  |  |
|  |  | *Porphyromonas* | 90,11 | 5,3 ± 8,26 | 94,51 | 4 ± 5,7 | NS | NS |
|  |  |  |  | 1,09  (0,2; 7,23) |  | 1,29  (0,11; 5,27) |  |  |
|  |  | *Alloprevotella* | 92,31 | 2,15 ± 3,24 | 94,51 | 1,83 ± 3,4 | NS | NS |
|  |  |  |  | 0,94  (0,26; 2,59) |  | 0,61  (0,2; 1,8) |  |  |
|  |  | *Tannerella* | 58,24 | 0,6 ± 1,78 | 67,3 | 0,2 ± 0,44 | NS | NS |
|  |  |  |  | 0,05  (0; 0,31) |  | 0,04  (0; 0,2) |  |  |
|  |  | *Rikenellaceae RC9 group* | 50,55 | 0,48 ± 1,39 | 39,56 | 0,17 ± 0,57 | NS | 0,03 |
|  |  |  |  | 0,03  (0; 0,25) |  | 0  (0; 0,07) |  |  |
|  |  | *Bacteroides* | 56,04 | 0,27 ± 0,77 | 41,76 | 0,75 ± 2,85 | NS | NS |
|  |  |  |  | 0,01  (0; 0,12) |  | 0  (0; 0,04) |  |  |
|  |  | *Rothia* | 50,55 | 0,17 ± 0,42 | 81,32 | 0,46 ± 0,91 | <0,001 | <0,001 |
|  |  |  |  | 0,01  (0; 0,13) |  | 0,1  (0,02; 0,38) |  |  |
|  |  | *Capnocytophaga* | 50,55 | 0,23 ± 1,24 | 63,74 | 0,18 ± 0,35 | NS | NS |
|  |  |  |  | 0,01  (0; 0,12) |  | 0,05  (0; 0,2) |  |  |
|  |  | *Bergeyella* | 21,98 | 0,03 ± 0,12 | 50,55 | 0,25 ± 1,66 | <0,001 | <0,001 |
|  |  |  |  | 0  (0; 0) |  | 0,01  (0; 0,06) |  |  |
| *Proteobacteria* | *Alphaproteobacteria* | *Allorhizobium-Neorhizobium-Pararhizobium-Rhizobium* | 98,90 | 8,77 ± 12,86 | 36,26 | 0,14 ± 0,65 | <0,001 | <0,001 |
|  |  |  |  | 3,85  (1,04; 9,42) |  | 0  (0; 0,03) |  |  |
|  |  | *Sphingomonas* | 96,7 | 2,3 ± 3,48 | 31,87 | 0,06 ± 0,27 | <0,001 | <0,001 |
|  |  |  |  | 1,06  (0,29; 2,52) |  | 0  (0; 0,02) |  |  |
|  |  | *Enhydrobacter* | 34,07 | 0,04 ± 0,12 | 81,32 | 0,41 ± 1,01 | <0,001 | <0,001 |
|  |  |  |  | 0  (0; 0,04) |  | 0,06  (0,02; 0,28) |  |  |
|  |  | *Paracoccus* | 19,78 | 0,02 ± 0,06 | 59,34 | 0,18 ± 0,41 | <0,001 | <0,001 |
|  |  |  |  | 0  (0; 0) |  | 0,02  (0; 0,12) |  |  |
|  | *Gammaproteobacteria* | *Haemophilus* | 87,91 | 3,02 ± 9,15 | 94,51 | 2,57 ± 4,22 | NS | NS |
|  |  |  |  | 0,55  (0,14; 1,97) |  | 0,68  (0,07; 3,66) |  |  |
|  |  | *Pseudomonas* | 82,42 | 0,19 ± 0,35 | 43,96 | 0,06 ± 0,13 | <0,001 | <0,001 |
|  |  |  |  | 0,06  (0,02; 0,22) |  | 0  (0; 0,04) |  |  |
|  |  | *Neisseria* | 58,24 | 0,39 ± 0,8 | 71,43 | 0,95 ± 2,89 | NS | NS |
|  |  |  |  | 0,04  (0; 0,32) |  | 0,07  (0; 0,5) |  |  |
|  |  | *Herbaspirillum* | 73,63 | 0,16 ± 0,32 | 34,07 | 0,02 ± 0,06 | <0,001 | <0,001 |
|  |  |  |  | 0,04  (0; 0,14) |  | 0  (0; 0,02) |  |  |
|  |  | *Escherichia-Shigella* | 56,04 | 0,08 ± 0,16 | 19,78 | 0,01 ± 0,03 | <0,001 | <0,001 |
|  |  |  |  | 0,02  (0; 0,07) |  | 0  (0; 0) |  |  |
|  |  | *Aggregatibacter* | 45,05 | 0,16 ± 0,37 | 59,34 | 0,3 ± 0,99 | NS | NS |
|  |  |  |  | 0  (0; 0,12) |  | 0,04  (0; 0,17) |  |  |
| *Fusobacteriota* | *Fusobacteriia* | *Fusobacterium* | 100 | 22,2 ± 24,56 | 97,8 | 21,55 ± 23,16 | NS | NS |
|  |  |  |  | 12,34  (4,47; 26,65) |  | 14,75  (3,35; 34,27) |  |  |
|  |  | *Leptotrichia* | 64,84 | 0,43 ± 0,97 | 76,92 | 0,86 ± 1,89 | NS | NS |
|  |  |  |  | 0,07  (0; 0,64) |  | 0,16  (0,01; 0,63) |  |  |
| *Actinobacteriota* | *Actinobacteriia* | *Actinomyces* | 79,12 | 0,32 ± 0,52 | 90,11 | 0,77 ± 1,22 | NS | 0,01 |
|  |  |  |  | 0,14  (0,03; 0,35) |  | 0,32  (0,05; 0,82) |  |  |
|  |  | *Microbacterium* | 85,71 | 0,21 ± 0,32 | 7,69 | 0 ± 0,02 | <0,001 | <0,001 |
|  |  |  |  | 0,07  (0,02; 0,23) |  | 0  (0; 0) |  |  |
|  |  | *Cutibacterium* | 72,53 | 0,14 ± 0,2 | 67,03 | 0,19 ± 0,75 | NS | NS |
|  |  |  |  | 0,06  (0; 0,17) |  | 0,02  (0; 0,12) |  |  |
|  |  | *Bifidobacterium* | 62,64 | 0,09 ± 0,22 | 57,14 | 0,14 ± 0,36 | NS | NS |
|  |  |  |  | 0,03  (0; 0,08) |  | 0,02  (0; 0,12) |  |  |
|  |  | *Corynebacterium* | 34,07 | 0,05 ± 0,12 | 65,93 | 0,27 ± 0,65 | <0,001 | <0,001 |
|  |  |  |  | 0  (0; 0,2) |  | 0,03  (0; 0,28) |  |  |
|  | *Coriobacteriia* | *Atopobium* | 67,03 | 0,44 ± 1,18 | 73,63 | 0,43 ± 1,14 | NS | NS |
|  |  |  |  | 0,12  (0; 0,40) |  | 0,06  (0; 0,26) |  |  |
|  |  | *Slackia* | 31,87 | 0,03 ± 0,08 | 53,85 | 0,06 ± 0,14 | <0,001 | 0,01 |
|  |  |  |  | 0  (0; 0,03) |  | 0,02  (0; 0,05) |  |  |
| *Spirochaetota* | *Spirochaetia* | *Treponema* | 76,92 | 1,74 ± 2,74 | 78,02 | 1,18 ± 2,32 | NS | NS |
|  |  |  |  | 0,34  (0,04; 2,36) |  | 0,17  (0,03; 1,08) |  |  |
| *Patescibacteria* | *Saccharimonadia* | *TM7x* | 51,65 | 0,2 ± 0,55 | 50,55 | 0,13 ± 0,4 | NS | NS |
|  |  |  |  | 0,01  (0; 0,13) |  | 0,01  (0; 0,09) |  |  |
| *Synergistota* | *Synergistia* | *Fretibacterium* | 50,55 | 0,59 ± 1,49 | 48,35 | 0,06 ± 0,16 | NS | 0,04 |
|  |  |  |  | 0,02  (0; 0,29) |  | 0  (0; 0,05) |  |  |

* NS – not significant

** Only the genera with abundance of $\boldsymbol{\geq}$0,01% are presented

*** Taxonomy of bacteria is subject to contiuous change. *Veillonella*, *Parvimonas* and *Peptostreptococcus* were recently transferred to new phylum *Bacillota* while *Haemophilus* and *Shingomonas* to new phylum *Pseudomonadota*.

**Table S5**. Incidence and abundance of species in pus and tonsils of PTA patients**

| **Phylum** | **Class** | **Genus** | **Species** | **Tonsils** | | **Pus** | | ***P* for incidence** | ***P* for abundance** |
| --- | --- | --- | --- | --- | --- | --- | --- | --- | --- |
|  |  |  |  | % positive | Mean ± SD | % positive | Mean ± SD |  |  |
|  |  |  |  |  | Median  (Q1; Q3) |  | Median  (Q1; Q3) |  |  |
| *Firmicutes* | *Bacilli* | *Streptococcus* | *S. mitis* | 82,42 | 2,09±4,47 | 73,63 | 5,21±9,42 | NS* | NS |
|  |  |  |  |  | 0,39  (0,04; 2,21) |  | 1,40  (0; 5,91) |  |  |
|  |  |  | *S. anginosus* | 72,53 | 0,87±1,99 | 89,01 | 3,64±12,03 | 0,008 | 0,01 |
|  |  |  |  |  | 0,14  (0; 0,80) |  | 0,37  (0,04; 1,48) |  |  |
|  |  |  | *S. pyogenes* | 65,93 | 10,02±19,98 | 70,33 | 16,54±28,17 | NS | NS |
|  |  |  |  |  | 0,16  (0; 7,88) |  | 0,15  (0; 24,50) |  |  |
|  |  |  | *S. parasanguinis* | 64,84 | 0,64±1,17 | 46,15 | 0,29±0,71 | 0,012 | 0,05 |
|  |  |  |  |  | 0,16  (0; 0,53) |  | 0  (0, 0,07) |  |  |
|  |  |  | *S. salivarius* | 50,55 | 0,25±0,63 | 74,73 | 0,50±1,06 | 0,001 | 0,005 |
|  |  |  |  |  | 0,01  (0; 0,19) |  | 0,07  (0; 0,45) |  |  |
|  |  | *Granulicatella* | *G. adiacens* | 62,64 | 0,26±0,52 | 80,22 | 0,48±0,86 | 0,014 | 0,01 |
|  |  |  |  |  | 0,03  (0; 0,32) |  | 0,17  (0,03; 0,47) |  |  |
|  |  | *Solobacterium* | *S. moorei* | 52,75 | 0,24±0,52 | 61,54 | 0,22±0,56 | NS | NS |
|  |  |  |  |  | 0,02  (0; 0,22) |  | 0,02  (0; 0,12) |  |  |
|  |  | *Bulleidia* | *B. extructa* | 36,26 | 0,21±0,50 | 54,95 | 0,43±0,99 | 0,012 | 0,031 |
|  |  |  |  |  | 0  (0; 0,12) |  | 0,02  (0; 0,27) |  |  |
|  | *Clostridia* | *Parvimonas* | *P. micra* | 83,52 | 1,84±2,69 | 90,11 | 3,41±4,94 | NS | NS |
|  |  |  |  |  | 0,92  (0,08; 2,55) |  | 1,37  (0,1; 5,21) |  |  |
|  |  | *Peptostrepto coccus* | *P. stomatis* | 72,53 | 0,63±1,11 | 76,92 | 1,66±4,11 | NS | NS |
|  |  |  |  |  | 0,20  (0; 0,76) |  | 0,12  (0,01; 0,98) |  |  |
|  |  | *Filifactor* | *F. alocis* | 60,44 | 0,60±0,85 | 62,64 | 0,59±1,37 | NS | NS |
|  |  |  |  |  | 0,08  (0; 0,92) |  | 0,05  (0; 0,57) |  |  |
|  |  | *Catonella* | *C. morbi* | 62,64 | 0,26±0,58 | 58,24 | 0,23±0,6 | NS | NS |
|  |  |  |  |  | 0,01  (0; 0,08) |  | 0,03  (0; 0,20) |  |  |
|  |  | *Eubacterium* | *E. brachy* | 52,75 | 0,39±0,81 | 72,53 | 1,15±2,38 | 0,009 | 0,12 |
|  |  |  |  |  | 0,02  (0; 0,37) |  | 0,09  (0; 1,05) |  |  |
|  |  | *Lachnoanaerobaculum* | *L. saburreum* | 35,16 | 0,08±0,22 | 52,75 | 0,09±0,23 | 0,025 | NS |
|  |  |  |  |  | 0  (0; 0,04) |  | 0,02  (0; 0,06) |  |  |
|  | *Negativicutes* | *Veillonella* | *V. atypica* | 73,63 | 0,85±1,50 | 72,53 | 0,51±1,10 | NS | NS |
|  |  |  |  |  | 0,18  (0; 0,99) |  | 0,13  (0; 0,41) |  |  |
|  |  |  | *V. dispar* | 59,34 | 0,84±1,98 | 60,44 | 0,38±0,69 | NS | NS |
|  |  |  |  |  | 0,06  (0; 0,56) |  | 0,05  (0; 0,40) |  |  |
|  |  |  | *V. parvula* | 34,07 | 0,29±0,78 | 58,24 | 0,61±1,23 | 0,002 | 0,002 |
|  |  |  |  |  | 0  (0; 0,06) |  | 0,05  (0; 0,44) |  |  |
|  |  | *Dialister* | *D. pneumosintes* | 64,84 | 0,64±0,88 | 69,23 | 1,03±1,72 | NS | NS |
|  |  |  |  |  | 0,20  (0; 1,06) |  | 0,12  (0; 1,31) |  |  |
|  |  |  | *D. invisus* | 51,65 | 0,31±1,33 | 48,35 | 0,10±0,31 | NS | NS |
|  |  |  |  |  | 0,01  (0; 0,21) |  | 0  (0; 0,03) |  |  |
|  |  | *Selenomonas* | *S. sputigena* | 54,95 | 0,23±0,55 | 47,25 | 0,09±0,28 | NS | 0,029 |
|  |  |  |  |  | 0,03  (0; 0,19) |  | 0  (0; 0,05) |  |  |
|  |  | *Megasphaera* | *M. micronuciformis* | 56,04 | 0,30±0,59 | 56,04 | 0,18±0,43 | NS | NS |
|  |  |  |  |  | 0,05  (0; 0,33) |  | 0,02  (0; 0,12) |  |  |
| *Bacteroidota* | *Bacteroidia* | *Prevotella* | *P. oris* | 86,81 | 3,96±7,59 | 91,21 | 5,06±8,99 | NS | NS |
|  |  |  |  |  | 0,42  (0,03; 3,33) |  | 0,77  (0,06; 6,03) |  |  |
|  |  |  | *P. melaninogenica* | 82,42 | 1,47±2,21 | 85,71 | 1,39±2,46 | NS | NS |
|  |  |  |  |  | 0,56  (0,06; 1,68) |  | 0,41  (0,07;1,64) |  |  |
|  |  |  | *P. pallens* | 67,03 | 0,54±1,15 | 65,93 | 0,32±0,75 | NS | NS |
|  |  |  |  |  | 0,06  (0; 0,50) |  | 0,05  (0; 0,24) |  |  |
|  |  |  | *P. histicola* | 63,74 | 0,49±1,05 | 51,65 | 0,32±0,96 | NS | 0,033 |
|  |  |  |  |  | 0,07  (0; 0,55) |  | 0,02  (0; 0,15) |  |  |
|  |  |  | *P. salivae* | 62,64 | 0,29±0,74 | 68,13 | 0,27±0,68 | NS | NS |
|  |  |  |  |  | 0,03  (0; 0,30) |  | 0,04  (0; 0,15) |  |  |
|  |  |  | *P. nigrescens* | 58,24 | 1,03±2,53 | 61,54 | 0,78±2,11 | NS | NS |
|  |  |  |  |  | 0,06  (0; 0,67) |  | 0,02  (0; 0,21) |  |  |
|  |  |  | *P. intermedia* | 52,75 | 0,88±1,91 | 50,55 | 0,73±2,37 | NS | NS |
|  |  |  |  |  | 0,01  (0; 0,47) |  | 0,01  (0; 0,24) |  |  |
|  |  | *Chryseo bacterium* | *C. hominis* | 96,70 | 2,96±4,20 | 17,58 | 0,04±0,24 | <0,001 | <0,001 |
|  |  |  |  |  | 1,26  (0,42; 3,17) |  | 0  (0; 0) |  |  |
|  |  | *Porphyro monas* | *P. endodontalis* | 70,33 | 3,13±6,01 | 75,82 | 2,63±4,80 | NS | NS |
|  |  |  |  |  | 0,15  (0; 3,31) |  | 0,10  (0,01; 2,27) |  |  |
|  |  |  | *P. pasteri* | 50,55 | 0,17±0,38 | 53,85 | 0,24±0,66 | NS | NS |
|  |  |  |  |  | 0,02  (0; 0,11) |  | 0,02  (0; 0,19) |  |  |
|  |  | *Alloprevotella* | *A. tannerae* | 63,74 | 0,69±1,18 | 69,23 | 0,89±3,00 | NS | NS |
|  |  |  |  |  | 0,09  (0; 0,58) |  | 0,05  (0; 0,36) |  |  |
|  |  |  | *A. rava* | 52,75 | 0,24±0,53 | 54,95 | 0,19±0,48 | NS | NS |
|  |  |  |  |  | 0,02  (0; 0,14) |  | 0,02  (0; 0,13) |  |  |
|  |  | *Tannerella* | *T. forsythia* | 51,65 | 0,56±1,77 | 47,25 | 0,09±0,25 | NS | NS |
|  |  |  |  |  | 0,02  (0; 0,22) |  | 0  (0; 0,08) |  |  |
|  |  | *Rothia* | *R. mucilaginosa* | 45.05 | 0,12±0,33 | 63,74 | 0,25±0,63 | 0,017 | 0,008 |
|  |  |  |  |  | 0  (0; 0,07) |  | 0,04  (0; 0,15) |  |  |
|  |  |  |  |  |  |  | 0,04  (0; 0,15) |  |  |
| *Proteobacteria* | *Alphaproteobacteria* | *Sphingomonas* | *S. faeni* | 96,70 | 2,28±3,44 | 5,49 | 0,04±0,25 | <0,001 | <0,001 |
|  |  |  |  |  | 1,05  (0,28; 2,52) |  | 0  (0; 0) |  |  |
|  |  | *Enhydrobacter* | *E. osloensis* | 28,57 | 0,04±0,12 | 63,74 | 0,38±1,00 | <0,001 | <0,001 |
|  |  |  |  |  | 0  (0; 0,02) |  | 0,04  (0; 0,17) |  |  |
|  | *Gammaproteobacteria* | *Haemophilus* | *H. parainfluenzae* | 80,22 | 1,16±2,71 | 89,01 | 1,72±3,35 | NS | NS |
|  |  |  |  |  | 0,16  (0,03; 0,72) |  | 0,26  (0,03; 1,97) |  |  |
|  |  | *Pseudomonas* | *P. fluorescens* | 53,85 | 0,08±0,14 | 21,98 | 0,02±0,06 | <0,001 | <0,001 |
|  |  |  |  |  | 0,02  (0; 0,09) |  | 0  (0; 0) |  |  |
| *Fusobacteriota* | *Fusobacteriia* | *Fusobacterium* | *F. nucleatum* | 89,01 | 6,80±8,77 | 85,71 | 7,78±13,79 | NS | NS |
|  |  |  |  |  | 2,64  (0,24; 10,35) |  | 2,11  (0,14; 8,77) |  |  |
|  |  |  | *F. necrophorum* | 82,42 | 14,62±26,38 | 74,73 | 13,28±23,19 | NS | NS |
|  |  |  |  |  | 0,22  (0,04; 15,68) |  | 0,12  (0; 20,46) |  |  |
| *Actinobacteriota* | *Actinobacteriia* | *Actinomyces* | *A. odontolytica* | 48,35 | 0,09±0,21 | 70,33 | 0,24±0,51 | 0,004 | 0,001 |
|  |  |  |  |  | 0  (0; 0,09) |  | 0,07  (0; 0,20) |  |  |
|  |  | *Micro bacterium* | *M. testaceum* | 84,62 | 0,20±0,32 | 1,10 | 0±0,02 | <0,001 | <0,001 |
|  |  |  |  |  | 0,07  (0,02; 0,23) |  | 0  (0; 0) |  |  |
|  |  | *Cutibacterium* | *C. acnes* | 70,33 | 0,13±0,19 | 63,74 | 0,18±0,71 | NS | NS |
|  |  |  |  |  | 0,05  (0; 0,16) |  | 0,02  (0; 0,11) |  |  |
|  | *Coriobacteriia* | *Atopobium* | *A. rimae* | 47,25 | 0,20±0,95 | 52,75 | 0,23±0,96 | NS | NS |
|  |  |  |  |  | 0  (0; 0,08) |  | 0,01  (0; 0,07) |  |  |
|  |  | *Slackia* | *S. exigua* | 31,87 | 0,03±0,08 | 53,85 | 0,06±0,14 | 0,004 | 0,013 |
|  |  |  |  |  | 0  (0; 0,03) |  | 0,02  (0; 0,05) |  |  |
| *Spirochaetota* | *Spirochaetia* | *Treponema* | *T. socranskii* | 50,55 | 0,22±0,44 | 47,25 | 0,10±0,19 | NS | NS |
|  |  |  |  |  | 0,02  (0; 0,23) |  | 0  (0; 0,12) |  |  |

* NS – not significant

** Only the species with abundance of $\boldsymbol{\geq}$0,01% are presented
